# Ethylene Enhances Seed Germination and Seedling Growth Under Salinity by Reducing Oxidative Stress and Promoting Chlorophyll Content *via* ETR2 Pathway

**DOI:** 10.3389/fpls.2020.01066

**Published:** 2020-07-16

**Authors:** Yue Wang, Pengfei Diao, Lingqi Kong, Ruonan Yu, Man Zhang, Tiantian Zuo, Yanyan Fan, Yiding Niu, Fang Yan, Hada Wuriyanghan

**Affiliations:** ^1^Key Laboratory of Forage and Endemic Crop Biotechnology, Ministry of Education, School of Life Sciences, Inner Mongolia University, Hohhot, China; ^2^State Key Laboratory of Reproductive Regulation & Breeding of Grassland Livestock, School of Life Sciences, Inner Mongolia University, Hohhot, China; ^3^Institute of Grassland Research, Chinese Academy of Agricultural Sciences, Hohhot, China

**Keywords:** alfalfa, salinity, ethylene, *MsETR2*, seed germination, seedling growth

## Abstract

Alfalfa (*Medicago sativa* L.) is an important forage, and salinity is a major stress factor on its yield. In this study, we show that osmotic stress retards alfalfa seedling growth, while ionic/oxidative stress reduces its seed germination. Ethylene treatment can recover the germination rate of alfalfa seeds under salt stress, while ethylene inhibitor silver thiosulfate exacerbates salt effects. ETH reduces the accumulation of MDA and H_2_O_2_ and increases POD activity. ETH and ACC improve the salt tolerance of alfalfa by increasing proline content under salt stress. In contrast, STS inhibits alfalfa seed germination by reducing POD activity. NaCl treatment reduces chlorophyll content in alfalfa leaves, while ETH and ACC can increase the chlorophyll content and promote seedling growth. ETH promotes the growth of alfalfa in saline condition by reducing the expression of *MsACO* and *MsERF8* genes, while increases its germination rate by upregulating *MsERF11* gene. Silencing of *MsETR2*, a putative ethylene receptor gene in alfalfa, abolishes ethylene triggered tolerance to salt stress. In summary, we show that ethylene improves salt tolerance in alfalfa *via MsETR2* dependent manner, and we also analyze the regulatory mechanism of ethylene during germination of alfalfa seeds under salt stress.

## Introduction

Soil salinity is one of the most common stresses that affect plant growth and development. Saline soil and alkaline soil often exist together in the actual environment, so they are collectively called saline and alkaline soil (Rengasamy, 2010). Most plants cannot grow normally in saline and alkaline soil environment, and medium salt concentration (100 mM NaCl) is sufficient to cause a sharp decline in most crop yields (Cheeseman, 2015). Plants mainly undergo two stages of rapid osmotic stress and slow ionic/oxidative stress (Munns and Tester, 2008). When the salt content in soil exceeds the threshold, it will cause higher water potential in the seed than that in the soil, thus inhibiting the water absorption of the seed from the external environment. Without sufficient water content in the seed, its germination and growth will be inhibited. Saline soil contains high concentration of metal ions, therefore the toxic effect of metal ions on seeds is another reason for inhibiting seed germination (Zhang et al., 2011).

Seed germination is the critical stage in plant growth and therefore directly affects the final yield of plants. In order to maintain growth and yield, plants adapt to abiotic stresses *via* specific tolerance mechanisms (Ibrahim et al., 2016). There are three main mechanisms of salt tolerance in plants, namely osmotic stress tolerance, maintenance of ion balance and reduction of Na^+^ concentration in cytoplasm (Deinlein et al., 2014). Abiotic stresses including salt stress are associated with the formation of reactive oxygen species (ROS), such as superoxide anion radicals (O_2_^−^), hydrogen peroxide (H_2_O_2_) and hydroxyl radicals (OH^-^) (Apel and Hirt, 2004). These are highly active molecules which cause oxidative damage to cells and biomolecules (Miller et al., 2010). Plants produce osmotic regulators in saline environment, and scavenge ROS species by their antioxidant enzyme activity. Both of them can alleviate the damage caused by salt stress. Antioxidants (ROS scavengers) include the enzymes such as catalase (CAT), peroxidase (POD), superoxide dismutase (SOD), ascorbate peroxidase (APX) and glutathione reductase (GR), as well as non-enzymatic molecules such as ascorbic acid (AA), glutathione (GSH), etc. Malondialdehyde (MDA) is one of the products of salt stress which damage the cell membrane of plants. Cellular content of MDA will increase under stress condition, while SOD can prevent or interrupt this damage (Neelam prabha et al., 2015). Proline (Pro) is a water-soluble amino acid that exists in plants in a free state. The accumulation of Pro reduces the water potential of cells and prevents toxic ions from entering cells (Rady et al., 2019). Multiple mechanisms for maintaining Na^+^/K^+^ balance in high salinity environments have also been reported, such as reducing net Na^+^ uptake from roots and reducing Na^+^ load in xylem (Zhu, 2003), transpiring Na^+^ from xylem sap to root (Tomoaki et al., 2010), accumulating Na^+^ in vacuoles to avoid cytoplasmic toxicity (Amtmann and Sanders, 1998), etc.

Seed germination is a complex process, and it is sensitive to many hormones and environmental factors (Finch Savag and Leubner Metzger, 2010). ABA inhibits seed germination, while ethylene and GA alleviate the inhibition of ABA on seed germination (Verma et al., 2016). NO is an important regulator in response to salt stress and may play a role in the downstream pathway of ABA. These signaling molecules and their downstream signaling components play an important role in salt tolerance in plants (Le et al., 2013). As a gaseous hormone, ethylene can regulate many aspects of plant growth and developmental processes, including seed germination, root hair formation, senescence, fruit ripening, etc. (Corbineau et al., 2014; Khan et al., 2017). 1-Aminocycloheptane-1-carboxylic acid (ACC) synthase (ACS) catalyzes the conversion of S-Adenosyl Methionine (SAM) to ACC, which is then oxidized by ACC oxidase (ACO) to produce ethylene (Larsen, 2015). Ethylene was reported to participate in plant salt stress tolerance. For example, increases in endogenous ethylene and ACC treatment can overcome the inhibition of salt stress on *Arabidopsis thaliana* seed germination (Divi et al., 2010). Silva et al. showed that NaCl could reduce seed germination rate and ethylene synthesis by increasing ABA concentration (Silva et al., 2014). Ethylene supply can effectively mitigate the inhibitory effects of salt stress on cucumber seed germination, and alleviate salt stress by regulating the synthesis and removal of ROS (Wang et al., 2011). In salt-stressed callus of *A. thaliana*, hydrogen peroxide can stimulate ethylene release, and ACC can enhance the production of hydrogen peroxide (Wang et al., 2010), which indicates that ethylene and hydrogen peroxide play as self-enhancing signal molecules in the feedforward loop (Soo Jin et al., 2010). Ethylene improves plant salt tolerance by accelerating exchange of K^+^ and Na^+^ across the membrane (Yang et al., 2013). In contrary to its ameliorative effects on salt stress, ethylene was also reported to play negative role in salt tolerance in some plant species including rice and tomato (Albacete et al., 2009; Tao et al., 2015). This indicated that ethylene involvements in salt stress response varied in different plant species.

Alfalfa (*Medicago sativa* L.) is a perennial leguminous plant with well-developed rhizomes. Because of its high protein content and good palatability, alfalfa is widely planted all over the world (Singer et al., 2018). In the present study, we showed that ethylene could ameliorate salt stress on different alfalfa varieties regardless of their sensitivity level, especially in terms of seed germination. The physiological and biochemical mechanisms underlying salt stress responses in alfalfa and involvement of ethylene in this process were investigated. While common mechanism was discovered, specific mechanism might exist in a certain variety. We also showed that ethylene improvement of salt tolerance in alfalfa was mediated *via MsETR2*, a putative ethylene receptor gene in alfalfa.

## Materials and Methods

### Seed Treatment, Determination of Germination Rate and Growth Parameters

Alfalfa seeds were surface-sterilized for 5 min in 75% ethanol, rinsed with ddH_2_O for three times and dried on filter paper at room temperature. One hundred alfalfa seeds were placed neatly in Petri dishes (9.0 cm diameter) containing two layers of filter paper soaked with 5 ml treatment solution at 2°C in illuminating incubator. The photoperiod was 16 h light and 8 h dark, and the luminous flux density was 40 μmol/m^2^·s. The effects of chemicals on seed germination under 250 mM NaCl were investigated by treating the seeds with solutions containing varying concentrations of ETH, ACC, STS (Silver thiosuifate) or combined solutions of them. The seed is regarded as being germinated when the radicle breaks through the seed coat. On the 10th day, the number of dead seeds, hard seeds, abnormal seedlings and the number of non-germinated seedlings were counted. Germination Rate = (Germinated seeds/Total seeds) * 100%. The shoot and root length were measured at the same time. Fresh weight was measured and then seedlings were put in an aluminum box, dried at 65°C in the oven for 1 h to constant weight, put in the dryer for 15 min, weighed and recorded. Relative water content = [(Fresh weight − Dry weight)/Fresh weight] * 100%.

### Determination of Pro, MDA, H_2_O_2_ Content and POD Activity

Seeds were exposed respectively to various combinations of solutions with 0.5 mM ETH, 10 μM ACC and 15 μM STS together with 250 mM NaCl. Fifth day seedling (0.1 g) was used as experimental material. The Pro, MDA, H_2_O_2_ content and POD activity were detected by different detection kits (Suzhou Keming Biotechnology Co., Ltd, China) according to manufacturer’s instructions.

### Determination of Na^+^ Content

Fifth day seedling (0.1 g) was used as experimental material. The fresh plants were washed three times with ddH_2_O, the surface moisture of the seedlings was blotted with filter paper, and then packed into paper bags. Samples were put into the oven and dried at 80°C until constant weight. 0.01 g dried sample was loaded into a 2 ml centrifuge tube, crushed with a high-throughput tissue crusher (50 Hz, 30 s, twice) added a steel ball. Add 1.8 ml 0.5 M HCl to each centrifugal tube and shake vigorously. The samples were incubated in shaker at 37°C for two days, and Na^+^ content was detected by atomic absorption spectrophotometer (4530F, Yuyang Industrial Co., Limited, China).

### Determination of Ethylene Content

Forty alfalfa seeds were exposed to 1 ml treatment solutions for 5 d in 10 ml vials. Five milliliters of the headspace was taken from the vials, and 1 ml of it was injected into a gas chromatograph equipment (GC-2014C). Chromatographic column: PEG-20M; Detector: FID; Carrier gas: N_2_; Gas pressure: 50 Kpa; Air: 45 Kpa; Diversion speed: 30 ml/min; Gasification chamber temperature: 200°C; Chromatographic column temperature: 45°C; Detector temperature: 220°C.

### DAB Staining

The seedlings were placed in a needle tube containing 1 mg/ml DAB solution. Seedlings were vacuumed to settle at the bottom of the solution, transferred to 50 ml conical flasks and stained at room temperature for 24 h. Seedlings were removed from DAB solution and put into 100 ml conical flasks. Seedlings were sealed with absolute ethanol and were decolorized in a miniature circular shaker at room temperature of 70 rpm for 24 h. Ethanol was replaced several times during the decolorization. Seedlings were removed from absolute ethanol, placed in 50 ml centrifuge tubes and ddH_2_O were added to the tubes. Finally, the seedlings were observed by a stereo microscope (Nikon, SMZ18).

### Pot Culture Experiment and Determination of Growth Parameters

Alfalfa seeds were germinated for one week in ddH_2_O and the seedlings were transplanted into soil. After the seedlings were grown for one week, they were irrigated with the treatment solutions. At 50th day post treatments, representative individuals from each group were photographed. Twenty individuals were measured for branch number, fresh weight and dry weight. Fifty leaves were randomly selected under each treatment, and the relative content of chlorophyll in leaves was detected by hand-held chlorophyll analyzer (REnQ-IV).

### RNA Extraction and qRT-PCR Analysis

For germination experiments, seeds were exposed respectively to various combinations of solutions with 0.5 mM ETH, 10 μM ACC and 15 μM STS together with 250 mM NaCl. Fifth day seedling was used as experimental material. For hairy root transformation experiments, the root tissues were collected and immediately frozen in liquid nitrogen and stored at −80°C for RNA extraction. First-strand cDNA synthesis was performed using Fermentas reverse transcription kit (HisScript^®^ II 1st Strand cDNA Synthesis Kit (+gDNA wiper) Vazyme, China). qPCR was performed according to the manufacturer’s instructions (TransStart^®^ Tip Green qPCR SuperMix, Quanshijin, China). The relative expression was calculated by using the 2^−ΔΔCt^ values with alfalfa *actin* as an internal standard. For qPCR experiments, three biological replicates were performed. Primer sequences are listed in the **Table S1**.

### Subcellular Localization of MsETR2 and *MsETR2*-*RNAi* Vector Construction for Transient Expression in Alfalfa

*MsETR2* cDNA encoding N-terminal 1-517aa protein fragment was ligated into pEarleyGate103-SL vector for analysis of subcellular localization. The recombinant vector was transformed to *Agrobacterium tumefaciens* strain GV3101, infiltrated into *N. benthamiana* leaves, and GFP signal was visualized at 36 hpi. Interference sequence for *MsETR2* (Unigene ID number: Medtr4g068740_ETR_EDR1) was cloned from alfalfa variety Juneng 418Q cDNA with primers (forward: 5’-GGAATTCAAGAACTGGAACATTCTGGAAGATCGTCCGTTTATGCCTC-3’ and reverse: 5’-CGGGATCCTGTGCCTACAGACCCCGTA-3’). The *MsETR2* interference sequence was ligated into the entry vector pENTR with *EcoR* I /*BamH* I and was subcloned into the binary vector pKGWRR-RFP using Gateway (Gateway^®^ LR Clonase™ II Enzyme Mix, Thermo, USA) recombination technology to obtain the interference vector pKGWRR-RNAi-*MsETR2*. The vector was then transformed into *Agrobacterium* strain Arqual1 for following experiment.

### Hairy Root Transformation of Alfalfa

Alfalfa (Juneng 418Q) seeds were sterilized with concentrated sulfuric acid for 5 min and then washed five times with sterile ddH_2_O, and immersed in a 10% sodium hypochlorite solution for 2 min, then rinsed five times with sterile water and soaked with sterile water for 5 h. The soaked seeds were transferred to a sterile filter paper and allowed to dry on the surface. The seeds were evenly spread in 0.8% FS sterilized solid medium, and grown the incubator (25°C, dark condition) for 3 days. The seed coat were removed with sterile forceps and then cut off the roots tip with a sterile knife by about 3 mm. The roots were clamped with forceps and soaked with the *Agrobacterium* solution at the wound site, and put in a 1.5% FS sterilized solid medium in square petri dish (15 × 15 cm). The seedlings were grown under greenhouse with a 16 h light (25°C ± 2°C)/8 h dark (18°C ± 2°C) photoperiod and 80% relative humidity for about three weeks. Transgenic seedlings were confirmed by appearance of RFP signal under stereo microscope (Nikon, SMZ18). The positive seedlings were then confirmed by qPCR experiment for *MsETR2* knockdown. The seedlings were transferred to a soil pot with perlite and vermiculite (1:1) in the greenhouse (25°C, 16 h light/22°C, 8 h dark). After one week, seedlings were treated with 137 mM NaCl or simultaneous addition of 0.5 mM ETH. Root tissue at 7 dpi was collected for expression analysis of ethylene related genes, and the growth parameters and chlorophyll content was measured at 10 dpi.

### Statistical Analysis

All experiments included at least three biological repeats, each expressed as mean ± standard error. The Excel 2010 software was used to analyze the data, and statistical variance was analyzed with SPSS 19.0 software.

## Results

### Salt Stress Negatively Affects Seed Germination and Seedling Growth of Different Alfalfa Varieties

**Figure 1:**
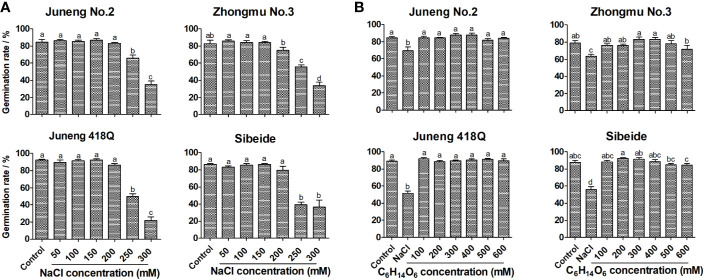
Effect of salt stress **(A)** and mannitol treatment **(B)** on germination rates of different alfalfa varieties. Control: Water; NaCl: 50, 100, 150, 200, 250, 300 mM. Mannitol: 100, 200, 300, 400, 500, 600 mM. For each replicate, one hundred alfalfa seeds were surface-sterilized, placed neatly in Petri dishes containing two layers of filter paper soaked with 5 ml treatment solution at 2°C in illuminating incubator. The seed is regarded as being germinated when the radicle breaks through the seed coat. On the 10th day, the germination rates were measured. Germination Rate = (Germinated seeds/Total seeds) * 100%. Error bars show the SEM between biological replicates performed (n = 4) and Duncan’s multiple range test was performed between samples in different groups.

To assess the effects of salt stress and also to evaluate the salt sensitivity of different alfalfa cultivars, germination rates were determined on 10th day upon exposure to varying concentrations (50–300 mM) of NaCl solution. While ≤200 mM NaCl showed no negative effects, 250 mM NaCl significantly reduced the germination rates of four alfalfa varieties. Under 300 mM NaCl, alfalfa seed germinations were severely inhibited, and the germination rates were decreased to 10–40% of the basal level. Therefore, the optimal concentration for NaCl treatment was chosen at 250 mM in all of the later experiment unless otherwise stated. Under 250 mM NaCl stress, the germination rates of Juneng No.2, Zhongmu No.3, Juneng 418Q and Sibeide were decreased by 22, 27, 42 and 47% compared with the control NaCl-free group respectively (**Figure 1A**). Therefore, Juneng No.2 was selected as salt-tolerant variety, Zhongmu No.3 was selected as moderate salt-tolerant variety, and Juneng 418Q and Sibeide were selected as salt-sensitive varieties for this study. As regards to the seedling growths, salt stress non-selectively reduced the root and shoot lengths of all four varieties in a concentration dependent manner (**Supplementary Figures 1A, B****)**.

In the initial period of salt treatment, plants mainly undergo two kinds of stresses: rapid osmotic stress and slow ionic/oxidative stress. In order to clarify the salt response mechanism of alfalfa, germinating alfalfa seeds were treated with mannitol solutions isotonic with NaCl concentrations. Mannitol treatment mimics osmotic stress as it mainly causes water loss in plant cells. Treatment with mannitol, even with as high concentration as 600 mM, did not decrease germination rates of four alfalfa varieties to the salt stress level (**Figure 1B**). Therefore, osmotic stress and dehydration are not the main reason for the decline of germination rate of alfalfa seeds by salt stress, while ionic/oxidative stress might be the reason. Although mannitol treatment did not influence germination rates, it negatively impacted seedling growth by reducing the lengths of both shoot and especially root in a dose-dependent manner, which was the same with NaCl treatment (**Supplementary Figures 2A, B****)**. Both NaCl and mannitol treatment decreased water contents in shoot and root, and mannitol triggered water loss was concentration dependent (**Supplementary Figure 3**). The results demonstrated that osmotic stress might be the reason for seedling growth retardation, while it did not affect germination rates.

### Exogenous Ethylene Alleviated Salt Inhibition on Alfalfa Seed Germination

Ethephon (ETH) is a synthetic substance with the same physiological function as ethylene, and ETH is generally used to substitute ethylene. Therefore, we treated alfalfa seeds under salt stress with ETH to explore the role of ethylene. As shown in **Supplementary Figure 4A**, addition of as low as 0.5 mM ETH could fully alleviate the inhibitory effects of NaCl treatment in terms of the germination rate. Although ethylene can increase the germination rate of alfalfa seeds under salt stress, it inhibits the growth of seedlings may be because of the ethylene-triggered triple responses (**Supplementary Figures 5A, B****)**. To confirm this, we used ETH to treat germinating alfalfa seedlings without NaCl, and we showed that ETH inhibited both root and shoot growths in a concentration dependent manner (**Supplementary Figures 6A, B****)**. The water content in shoot and root under salt stress decreased compared with the control group, and the addition of ETH in NaCl solution did not increase the water content, indicating that ETH did not alleviate the inhibition of salt stress by increasing the water content (**Supplementary Figure 7**).

ACC is the precursor of ethylene biosynthesis, and it can be converted into ethylene *via* the oxidation by ACO. As the same with ETH, ACC treatment alleviated salt triggered decline of alfalfa seed germination (**Supplementary Figure 4B**). Although exogenous addition of ETH did not alleviate the inhibitory effects on seedling growth (**Supplementary Figure 5**), exogenous addition of ACC could partially alleviate the inhibition of seedling growth under salt stress (**Supplementary Figure 8**). If ACC only participates in the ethylene biosynthesis pathway, the inhibition of alfalfa seedling growth will not be alleviated by ACC. It indicates that ACC might participate in other metabolic pathways instead of ethylene biosynthesis pathway, at least to some extents, to improve alfalfa seed germination rate and therefore also promote the seedling growth under salt stress. As the same with ETH, ACC treatment did not alleviate the inhibition of salt stress by increasing the water content of plant cells (**Supplementary Figures 9A, B****)**.

STS (silver thiosulfate) is a competitive inhibitor of ethylene signal transduction pathway. Ethylene binds to ethylene receptor to trigger a series of downstream reactions. As a competitive antagonist, STS binds ethylene receptor and reduces the binding rate of ethylene to its receptor. As shown in **Supplementary Figure 4C**, STS treatments exacerbated the inhibitory effects of NaCl on alfalfa seed germination in a concentration dependent manner. The inhibitory effects of STS on alfalfa seed germination should be conferred *via* its competition with endogenously preexisting ethylene, this supported our assumption that endogenous ethylene had counteracting role to salt toxicity in terms of seed germination. Although STS can strengthen the degree of stress and inhibit the germination of alfalfa seeds, it can promote the growth of alfalfa seedlings, which was opposite to ETH, further supporting the speculation that STS affects plant growth by inhibiting ethylene triggered triple responses in alfalfa (**Supplementary Figures 10A, B****)**. In summary, ETH and ACC treatments recovered seed germination rate, while STS treatment further aggravated the effects of salt stress. Taken together, above mentioned data strongly suggest that ethylene plays positive role on seed germination of alfalfa under salt stress.

**Figure 2:**
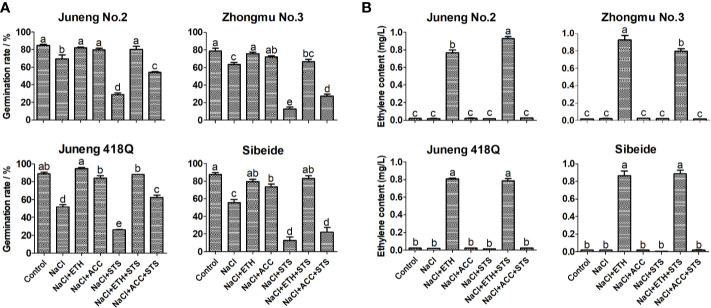
Effects of combined treatment on germination rates **(A)** and ethylene contents **(B)** of different alfalfa varieties under salt stress. Control: Water; NaCl: 250 mM; ETH: 500 μM; ACC: 10 μM; STS: 15 μM. Error bars show the SEM between biological replicates performed (n = 4) and Duncan’s multiple range test was performed between samples in different groups. For ethylene content measurement, forty alfalfa seeds were exposed to 1 ml treatment solutions for 5 d in 10 ml vials. 5 ml of the headspace was taken from the vials, and 1 ml of it was then injected into a gas chromatograph equipment (GC-2014C) for determination of ethylene content.

**Figure 3:**
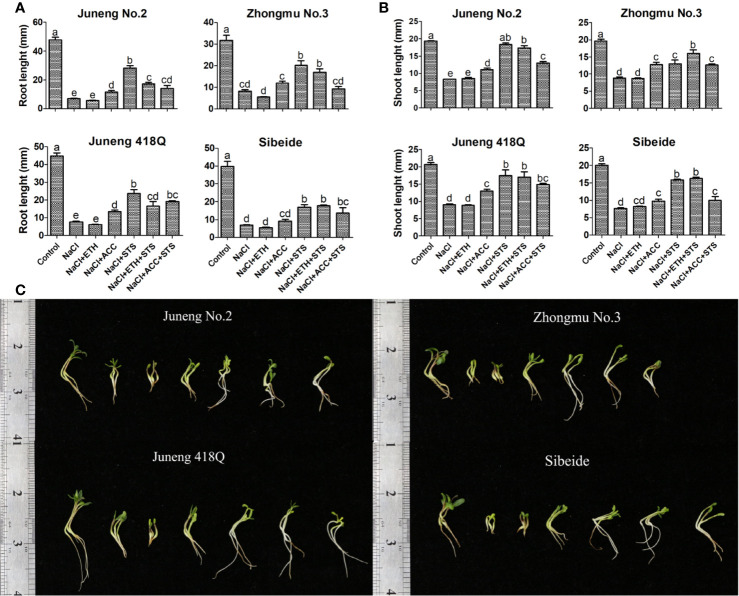
Effects of combined treatment on seedling growth of different alfalfa varieties under salt stress. **(A)** Root length. **(B)** Shoot length. **(C)** Phenotype of representative seedlings. Control: Water; NaCl: 250 mM; ETH: 500 μM; ACC: 10 μM; STS: 15 μM. On the 10th day, the root and shoot lengths were measured. Error bars show the SEM between biological replicates performed (n = 4) and Duncan’s multiple range test was performed between samples in different groups. Representative seedlings were photographed on the 10th day. Scale bar was shown on the left.

To further demonstrate the role of ethylene, combined treatments of ETH or ACC, and STS, together with NaCl, were performed in the following experiments. Addition of STS partly abolished the ameliorative effects of ACC treatment, while addition of ACC partly recovered STS induced decline in germination rate. In contrary to ACC, addition of STS did not compromise the effects of ETH, while addition of ETH fully recovered STS induced decline in germination rate. The possible reason for differential effects of ACC and ETH is that exogenous application of ETH competes over STS by binding to ethylene receptor as the amount of ethylene produced by exogenous application of ETH is much higher than the endogenously produced ethylene. Compared with ETH, exogenous application of ACC did not produce plenty of ethylene, therefore it may alleviate salt stress by another metabolic pathway (**Figure 2A**). The ethylene content released from germinating alfalfa seedlings under different treatments was determined by gas chromatography. Consistent with the above results, addition of ETH greatly increased ethylene release while addition of ACC did not raise ethylene release in alfalfa seedlings (**Figure 2B**). Therefore, the results here supported again that ACC did not mainly function through production of ethylene in alfalfa. As in previous experiments, ethylene produced by ETH has an effect of triple response on alfalfa seedling growth, while ACC treatment can alleviate the inhibition of salt stress on alfalfa seedling growth. However, as long as STS is added, the seedling growth of alfalfa is significantly higher than that of alfalfa under salt stress. The seedling length of alfalfa treated with NaCl + ETH + STS were significantly higher than those treated with NaCl + ETH. This indicated that STS could bind to ethylene receptor, resulting in the decrease of ethylene binding, alleviating the triple response of ethylene, thus promoting the growth of alfalfa seedlings (**Figures 3A–C**).

### Physiological Effects of Ethylene on Salt Treated Alfalfa Seedlings

**Figure 4:**
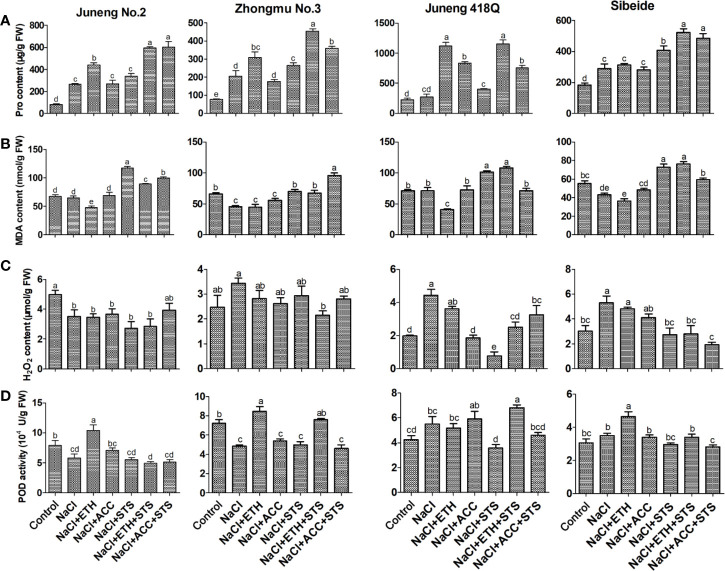
Physiological parameters of different alfalfa varieties after combined treatment with salt stress. **(A)** Proline content. **(B)** MDA content. **(C)** H_2_O_2_ content. **(D)** POD activity. Control: Water; NaCl: 250 mM; ETH: 500 μM; ACC: 10 μM; STS: 15 μM. On the 5th day, the physiological parameters were measured. Error bars show the SEM between biological replicates performed (n = 4) and Duncan’s multiple range test was performed between samples in different groups.

In order to examine the physiological effects of salt stress and the role of ethylene treatment in this process, the contents of Pro, MDA, H_2_O_2_ and POD activity were measured. Under salt stress, the *in vivo* Pro content increased, which was an indicator of plant resistance response against stress treatment. Addition of ETH further elevated the Pro content in most cases, while ACC treatment increased Pro content only in Juneng 418Q. Combined treatment of STS together with ETH or ACC induced Pro content in all varieties. In salt-sensitive variety Juneng 418Q the increase in Pro content was higher than those in three other varieties, indicating that Juneng 418Q is more sensitive to ethylene treatment. The results demonstrated that elevated Pro content might be the reason for ethylene mediated salt tolerance in some varieties especially like Juneng 418Q (**Figure 4A**).

MDA content represents the degree of oxidative damage on cell membrane system. Addition of ETH decreased MDA content in most cases, while addition of STS increased MDA level, demonstrating that ETH and STS participated in same regulatory pathway and they played opposite roles. Compared with this, ACC treatment did not change MDA level, suggesting that ACC alleviated salt stress by other mechanism instead of decreasing MDA level (**Figure 4B**).

In salt-tolerant cultivar Juneng No.2, the content of H_2_O_2_ decreases under salt stress and also together with the treatments such as ETH, ACC and STS. Therefore, the decrease of H_2_O_2_ content under salt stress may be the cause of salt tolerance of Juneng No.2. In moderate-sensitive cultivar Zhongmu No.3, there was no significant difference in the content of H_2_O_2_ after various treatments compared with that of the control group. In salt-sensitive varieties Juneng 418Q and Sibeide, the content of H_2_O_2_ increased after salt stress, and then decreased after treatments with ETH, ACC and STS. Taken together, different accumulation of H_2_O_2_ content under salt stress might be one of the reasons for salt tolerance variability in different alfalfa varieties used in our study here (**Figure 4C**).

In salt-tolerant cultivar Juneng No.2 and moderate-tolerant cultivar Zhongmu No.3, POD activity decreased after NaCl stress and increased after ETH treatment, demonstrating that ETH-mediated salt alleviation in these two varieties was due to the increases in POD activity. In salt sensitive varieties Juneng 418Q and Sibeide, POD activity decreased under STS treatment and slightly increased under ETH treatment respectively. Taken together, the POD activity of most of alfalfa varieties treated with ETH was significantly higher than that under salt stress, indicating that ETH mitigated the salt stress by elevating POD activity (**Figure 4D**). DAB (Diaminobenzidine) and H_2_O_2_ produce colored insoluble sediments under catalysis by POD. Therefore, DAB staining is the indicator of the status of oxidative stress, and it is due to the combined effects of POD activity and H_2_O_2_ content. As seen in **Supplementary Figure 11**, DAB staining results were consistent with the results of H_2_O_2_ content and POD activities.

Under salt stress, Na^+^ concentration would increase and plant growth would be retarded. The result from mannitol treatment of alfalfa showed that osmotic stress was not the main reason to reduce the germination rate of alfalfa, therefore the Na^+^ content in the stressed plants was determined. Na^+^ content in plants under salt stress was remarkably higher than that in control plants, while it was significantly decreased after exogenous addition of ETH and ACC in the alfalfa varieties except Juneng 418Q (**Supplementary Figure 12**). This indicated that ETH could alleviate salt stress inhibition on alfalfa seed germination by reducing Na^+^ content in alfalfa.

### Ethylene Related Genes Are Involved in Alfalfa Responses Toward Salt Stress

**Figure 5:**
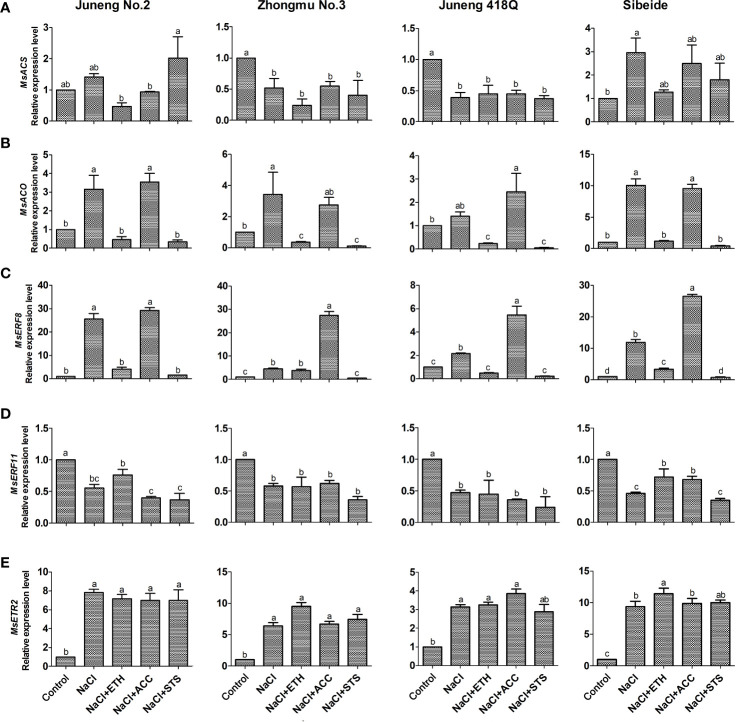
Expression analysis of ethylene related genes in different alfalfa varieties seedlings after combined treatment with salt stress. **(A)** *MsACS* expression. **(B)** *MsACO* expression. **(C)** *MsERF8* expression. **(D)** *MsERF11* expression. **(E)** *MsETR2* expression. Control: Water; NaCl: 250 mM; ETH: 500 μM; ACC: 10 μM; STS: 15 μM. Error bars show the SEM between biological replicates performed (n = 3) and Tukey’s multiple comparisons test was performed between samples in different groups. Relative expression levels of target genes are normalized against steady state levels of alfalfa *actin* gene.

In order to reveal molecular mechanisms underlying salt stress responses and ethylene mediated mitigatory effects, we assayed expression of four genes involving ethylene biosynthesis and downstream signal transduction by qRT-PCR. Alfalfa genome encoded 11 ACS homologs and 48 ACO homologs, and we did phylogenetic analysis to indicate their evolutionary relationships (**Supplementary Figures 13A, B****)**. We found 159 ERF homologs in alfalfa (Jin et al., 2019), and we classified them to 10 subfamilies (**Supplementary Figure 13C**). We selected *MsERF8* and *MsERF11* for expression analysis as they were reported to be involved in NaCl and ethylene responses in alfalfa (Chen et al., 2012a; Chen et al., 2012b). Expression of *MsACS* did not show regular patterns after salt stress and ethylene treatment **(****Figure 5A****)**. Expression of *MsACO* and *MsERF8* showed same regulatory patterns. Salt treatment induces expression of both *MsACO* and *MsERF8*, demonstrating that these two genes might be involved in salt responses in alfalfa. Addition of ETH abolished NaCl induction on *MsACO* and *MsERF8* expression, while addition of ACC did not change this induction **(****Figures 5B, C****)**. As MsACO catalyze the last step in ethylene biosynthesis, we hypothesize that ETH treatment triggers feedback inhibition on *MsACO* to down-regulate its expression. Expression of *MsERF8* showed same regulatory patterns upon salt stress and ethylene treatment, demonstrating that *MsERF8* might act downstream of ethylene transduction pathway and therefore plays important and specific roles in alfalfa responses to salt stress. The expression of *MsERF11* gene was significantly down-regulated by salt stress in all of the alfalfa varieties, while its expression was partly elevated by ETH treatment in Juneng No.2 and Sibeide. In contrary to ETH treatment, STS treatment generally further down-regulated the expression of *MsERF11* as compared with the salt stress alone. In conclusion, *MsERF11* gene participates in salt stress and might positively regulate alfalfa seed germination (**Figure 5D**).

### Ethylene Mitigates Salt Stress and Promotes Alfalfa Growth in Soil Culture

**Figure 6:**
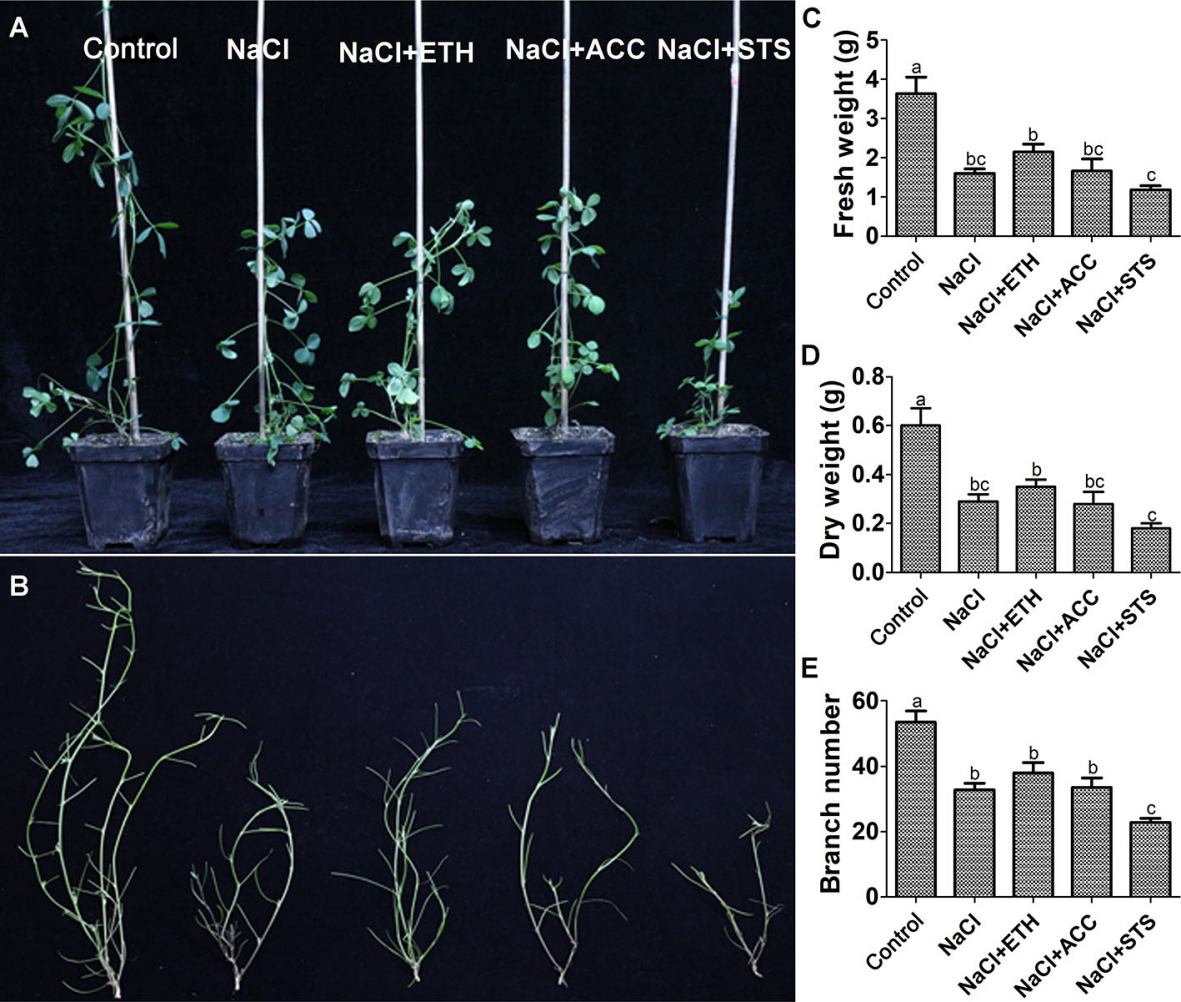
Growth related parameters of soil grown alfalfa seedlings upon combined treatment with salt stress. Control: Water; NaCl: 175 mM; ETH: 500 μM; ACC: 10 μM; STS: 15 μM. Alfalfa seeds were germinated for one week in ddH_2_O and the seedlings were transplanted into soil. After the seedlings were grown for one week, they were irrigated with the treatment solutions. At 50th day post treatments, representative individuals from each group were photographed. **(A)** The phenotype of representative seedlings from each treatment, scale bar was shown. **(B)** Branching phenotype of the seedlings, scale bar was shown. **(C)** Fresh weight. **(D)** Dry weight. **(E)** Branch number. n = 20.

**Figure 7:**
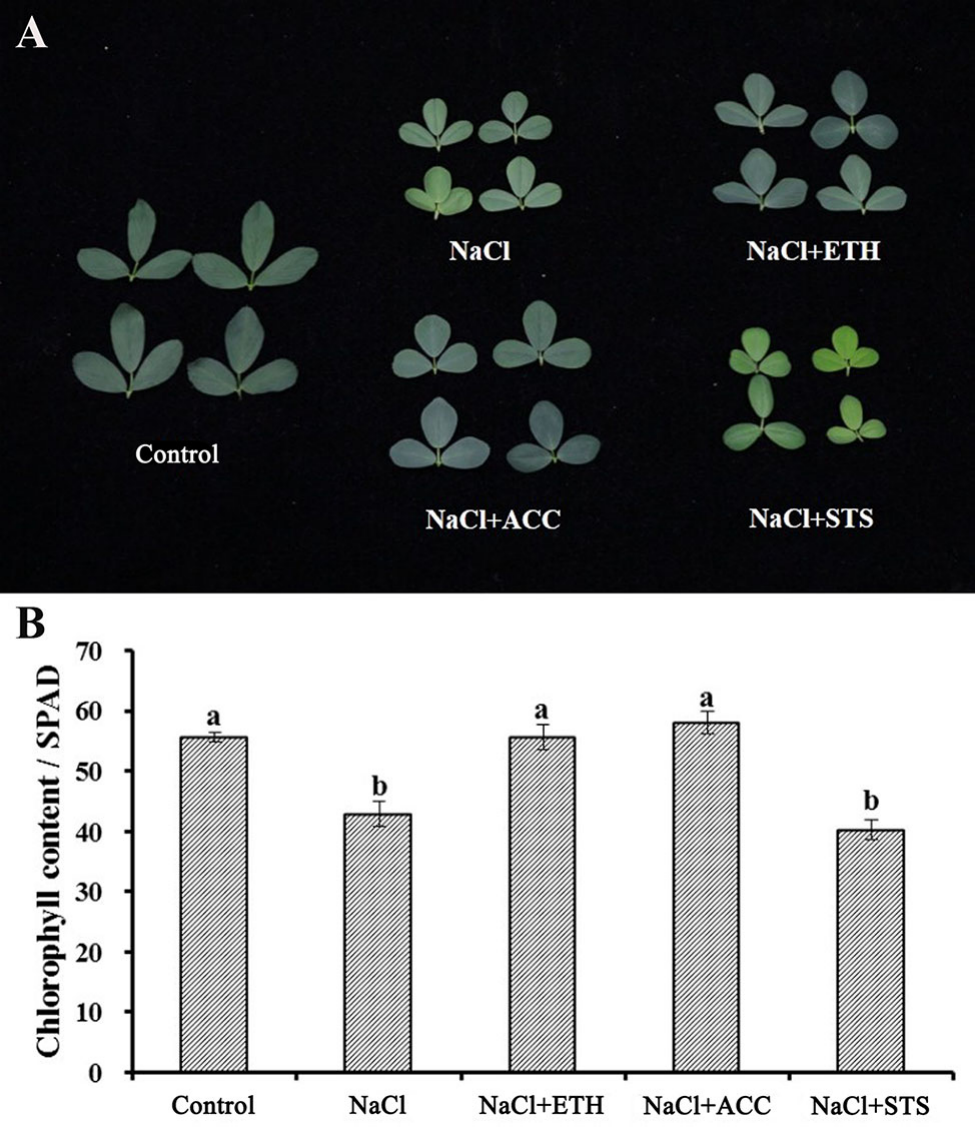
Leaf phenotype and chlorophyll content of soil grown alfalfa seedlings upon combined treatment with salt stress. **(A)** Phenotype of representative leaves. **(B)** Chlorophyll content. Control: Water; NaCl: 175 mM; ETH: 500 μM; ACC: 10 μM; STS: 15 μM. Alfalfa seeds were germinated for one week in ddH_2_O and the seedlings were transplanted into soil. After the seedlings were grown for one week, they were irrigated with the treatment solutions. At 50th day post treatments, fifty leaves were selected for each treatment and chlorophyll content was measured with a chlorophyll analyzer (REnQ-IV). Representative leaves from each treatment were photographed.

In all of the above experiments the plants were cultured in water culture dish. Therefore, we further investigated the role of ethylene on salt stress by soil cultured seedlings of salt sensitive Juneng 418Q variety. The growth parameters were determined upon ethylene related chemicals were added to salt irrigated alfalfa plants. The plant height of salt-stressed alfalfa seedlings significantly decreased, while ETH and ACC treatment did not alleviate this growth retardation. The fresh weight, dry weight and branch number of alfalfa seedlings all decreased significantly under salt stress. ETH treatment alleviated salt stress by increasing fresh weight, dry weight and branch number of alfalfa seedlings, while STS treatment further aggravated the growth inhibition by salt stress (**Figures 6A–E**). Salt stress decreased chlorophyll content and therefore also caused leaf yellowing of soil-cultured alfalfa seedlings. Both ETH and ACC treatments relieved salt stress by increasing chlorophyll contents, while STS treatment further enhanced salt stress by exacerbating the leaf yellowing phenotype (**Figures 7A, B****)**. Taken together, these data further demonstrated the alleviating effects of ethylene on salt stressed alfalfa growth by increasing chlorophyll contents.

### Knockdown of *MsETR2* Compromised Ethylene Mediated Resistance to Salt Stress in Alfalfa Plants

**Figure 8:**
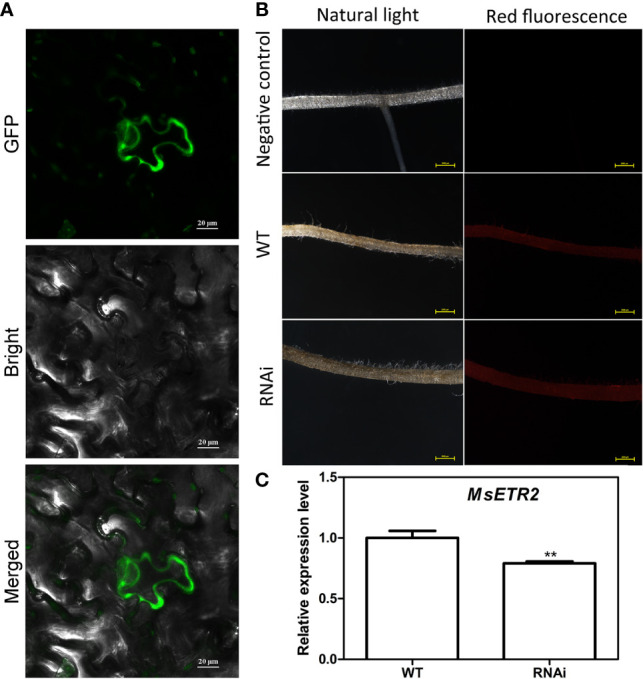
Analysis of MsETR2 subcellular localization and silencing of *MsETR2 via* hairy root transformation. **(A)** MsETR2 subcellular localization. GFP was observed by confocal microscopy (Zeiss, LSM710) under 635 nm red excitation light. **(B)** Confirmation of hairy root transformation by RFP detection. Left panel is the alfalfa hair root taken under natural light, and right panel is the alfalfa hair root photographed under green excitation light. **(C)** Expression analysis of *MsETR2*. For qRT-PCR experiment, the housekeeping gene *actin* was used as the reference. Data represent mean values for three independent biological replicates. Standard errors are indicated by vertical bars. Asterisks indicate statistical difference of the value at the < (**) levels as determined by t-test.

**Figure 9:**
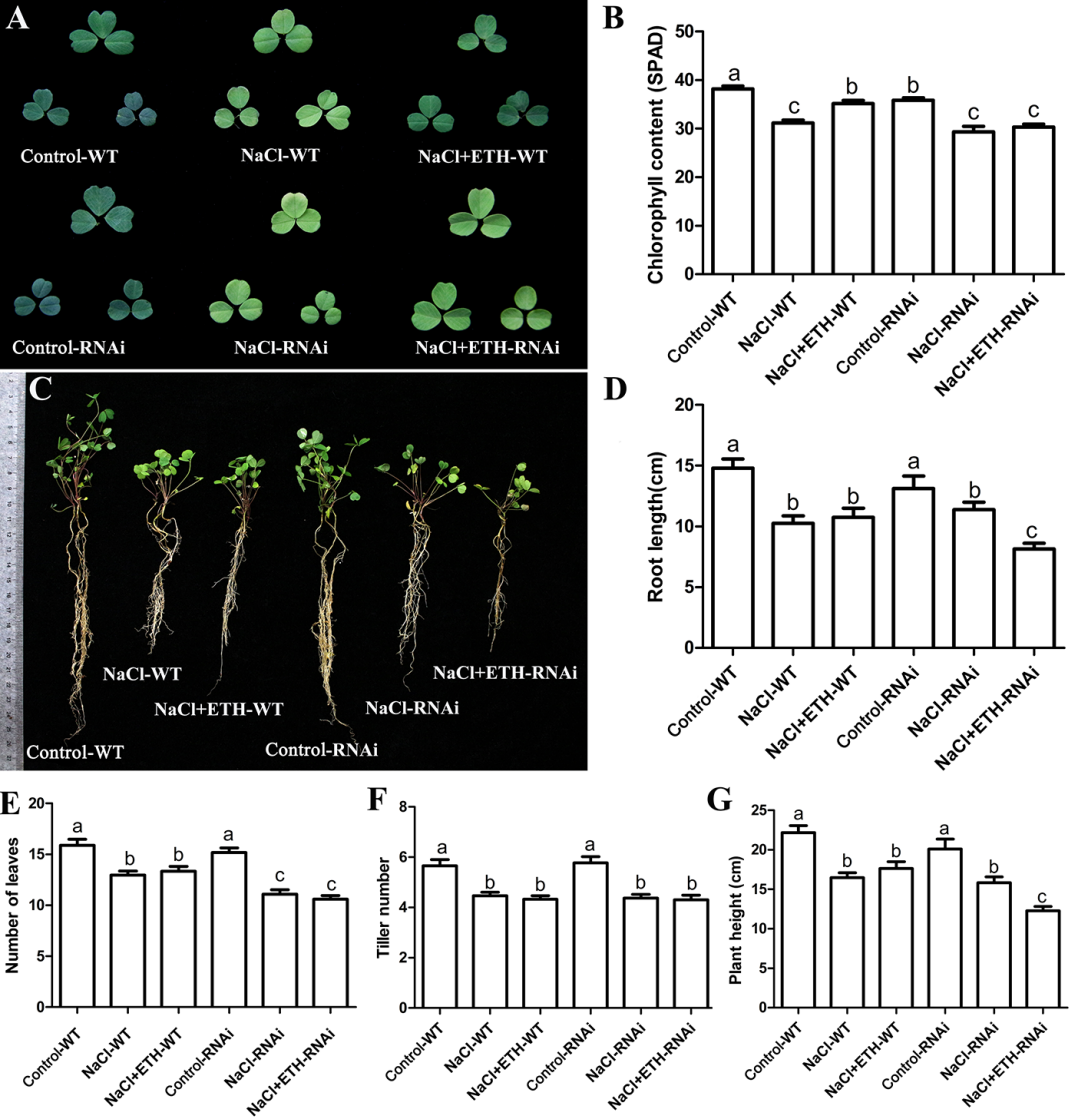
Response of *MsETR2* RNAi plants toward combined treatment with salt stress. **(A)** The phenotype of representative leaf after treatment. **(B)** Chlorophyll content (n = 45). **(C)** The phenotype of the whole plants after treatment. **(D)** Root length (cm) (n = 15). **(E)** Leaf number (n = 15). **(F)** Plant height (n = 15). **(G)** Tiller number (n = 15). WT, wild type plant; RNAi, *MsETR2*-silenced plant; Control, control water treatment; NaCl, salt stress treatment; NaCl + ETH, both NaCl and ethylene treatment. Error bars show the SEM between biological replicates performed (n = 3) and Tukey’s multiple comparisons test was performed between samples in different groups.

In order to further determine the alleviating role of ethylene on salt stressed alfalfa growth, we cloned *MsETR2*, a putative ethylene receptor gene, and investigated its involvement in ethylene-mediated salt tolerance in alfalfa. MsETR2 showed highest homology with *Zea mays* ethylene receptor 2 (**Supplementary Figure 14**). MsETR2 was localized on cell membrane, demonstrating that it might be an active ethylene receptor in alfalfa **(****Figure 8A****)**. Furthermore, *MsETR2* expression was induced by NaCl treatment in all four varieties, demonstrating its involvement in alfalfa salt responses (**Figure 5E**). *MsETR2* knockdown plants were generated by transformation of *MsETR2* specific RNAi construct *via* hairy root transformation method in salt sensitive Juneng 418Q variety (Spano et al., 1987). Transgenic lines were verified by detection of vector pKGWRR expressed RFP, and qPCR analysis indicated that the expression level of *MsETR2* was decreased in the RNAi lines (**Figures 8B, C****)**. Salt stress caused leaf yellowing and decrease in chlorophyll content. While ethylene treatment relieved the leaf yellowing phenotype of salt stress by increasing chlorophyll contents in control plants, it could not alleviate the negative effects of salt stress in *MsETR2* RNAi seedlings. Down-regulation of *MsETR2* also caused decrease in root length, number of leaves and plant height compared with wild-type plants under treatment with exogenous ETH in NaCl solution. Significant differences in number of tillers were observed between WT and RNAi lines neither with salt nor in the exogenous ETH in NaCl solution (**Figures 9A–G**). The results demonstrated that relieving effects of ethylene on salt stressed alfalfa plants were dependent on *MsETR2*. qPCR results indicated that the level of *MsACO*, *MsERF8* and *MtERF11* were all down-regulated in *MsETR2* RNAi plants after ETH treatment in NaCl solution, which was in accordance with the above observation and *MsETR2* might have feedback regulatory role on *MsACO* expression (**Supplementary Figures 15A–D**).

## Discussion

Salinization, one of the most common environmental factors, inhibits the normal growth of plants and affects the yield of crops and forages. Despite alfalfa being a medium salt-tolerant forage, high concentration of NaCl would also negatively affect its growth and yield. In the present study, we evaluated four alfalfa varieties for salt tolerance and identified tolerant (Juneng No.2), medium-tolerant (Zhongmu No.3) and sensitive (Juneng 418Q and Sibeide) varieties by seed germination index. Our result is in consistence with previous results for medium-tolerant varieties Zhongmu No.1 and Zhongmu No.3 (Ma et al., 2017; Lei et al., 2018), demonstrating the reliability of our experiments. Germination rate was reduced by NaCl stress but not by the isotonic mannitol treatment. Both NaCl and mannitol treatments inhibited the growth of alfalfa seedlings, but the water content under NaCl stress was significantly higher than that under isotonic mannitol treatment. In addition, Na^+^ content in alfalfa under NaCl stress was significantly higher than that in control group, indicating that osmotic stress in NaCl stress affected the growth of alfalfa seedlings, while ionic/oxidative stress affected the germination of alfalfa seeds. Our results are consistent with proteome analysis of alfalfa under salt and drought conditions (Ma et al., 2016). Ribeiro et al. found that ethylene played a key role in breaking dormancy and germination of stylosanthes seeds (Ribeiro and Barros, 2006), and Silva et al. also found that ethylene played a role in salt tolerance of stylosanthes (Silva et al., 2014). Our study showed that addition of ETH could elevate the ethylene content and increase the germination rate of alfalfa seeds under salt stress. The result indicated that ethylene played a crucial role in improving salt tolerance of alfalfa.

In order to adapt to saline and alkaline environment, plants would undergo a series of morphological, physiological and biochemical changes which are beneficial to plant growth. For example, increases in osmotic regulators like proline and antioxidant enzymes like POD or decreases in oxidative stress indicator H_2_O_2_ and lipid peroxidation indicator MDA in plants can reduce the damage to plant cell membranes caused by salt stress (Deinlein et al., 2014). Under NaCl treatment, proline contents were significantly higher than those of the control group, and the increase levels were positively correlated with salt tolerance degree of four alfalfa varieties. Moreover, ETH and ACC further increase proline contents in NaCl-stressed alfalfa, indicating that the above treatment can enhance the salt tolerance of plants by elevating proline level. MDA was the indicator for level of membrane lipid peroxidation and was reported to accumulate at higher levels in sensitive plants than in resistant plants (Shalata and Tal, 1998; Wu et al., 2003). Although different alfalfa cultivars varied in salt tolerance, alfalfa itself is relatively tolerant to salt stress than the sensitive plants like tobacco and *A. thaliana*. Our data also illustrated that MDA content did not change or even decreased in four alfalfa cultivars under salt stress, which were consistent with the data for relatively salt-tolerant alfalfa variety Zhongmu No.1 and salt-sensitive alfalfa variety Xingjiang Daye (Lei et al., 2018). ETH treatment significantly decreased the content of MDA, while STS treatment elevated MDA level in NaCl-stressed alfalfa. After NaCl stress, the content of H_2_O_2_ increases in salt-sensitive varieties Juneng 418Q and Sibeide, which was same with the result for alfalfa cultivar Magnum Salt (Lou et al., 2018). H_2_O_2_ content decreases after ETH or ACC treatment, which alleviates the degree of oxidative damage of cell membrane system and improves the salt tolerance of alfalfa to a certain extent. ETH could enhance the POD activity and salt tolerance of plants, while STS reduced the POD activity and further enhanced the stress. It is interesting to note that ACC can be converted into peroxidase in some instances (Acosta et al., 1991).

When plants were exposed to abiotic stresses, the expression of many growth and development-related genes, such as ethylene metabolism-related genes will change. Pei et al. found that root growth was inhibited after aluminum stress *via* up-regulating the expression of *MtACO* and *MtACS* genes in *Lotus japonicus* (Pei et al., 2007). In all of the four varieties tested, salt stress strongly induced *MsACO* expression. Considering the ameliorative effects of ethylene on salt stress in alfalfa from our data, we assume that alfalfa plants respond to salt stress by elevating *MsACO* expression. Treatment with both ETH and STS abolished salt induction on *MsACO* expression. We hypothesize that ETH treatment greatly increases ethylene content, thereby leading to feedback inhibition on *MsACO* expression. STS treatment block ethylene signal pathway, thereby also cause feedback suppression on *MsACO* expression. Our data are consistent with the feedback regulation of ethylene biosynthesis genes in tomato (Chang et al., 2008). In contrary to ETH and STS, ACC did not alter *MsACO* expression, further supporting the idea that ACC mainly did not function through conversion to ethylene in alfalfa, as was reported in the plant species like *Pyrus communis* (Ruan et al., 2000). *MsERF8* and *MsERF11*, two ethylene response factor encoding genes from alfalfa, were reported to be induced by salt stress and to enhance resistance to salinity after overexpression in tobacco or *A. thaliana* respectively (Chen et al., 2012a; Chen et al., 2012b). Expressions of *MsERF8* under NaCl, NaCl + ETH and NaCl + STS were induced by NaCl and suppressed by ETH or STS treatments. The results indicated that *MsERF8* might have functioned downstream of the ethylene signal transduction pathway and played important roles in ethylene mediated salt tolerance in alfalfa. Interestingly, NaCl induction on *MsERF8* expression was greatly enhanced by ACC treatment in relatively salt-sensitive Zhongmu NO.3, Juneng 418Q and Sibeide by 2–6 folds while was not changed in salt-tolerant Juneng No.2. The results illustrated that *MsERF8* gene was involved in ACC mediated salt tolerance in these three alfalfa varieties. In contrary to *MsERF8*, expressions of *MsERF11* were generally suppressed by NaCl in all of four alfalfa varieties, and this suppression was only partly recovered by ETH and ACC treatment in salt-sensitive variety Sibeide.

Ethylene was reported to enhance photosynthesis in wheat and mustard under salt stress (Khan, 2003; Nazar et al., 2014). Besides the ameliorative effects of ethylene on alfalfa seed germination under salt stress, ethylene also improved alfalfa growth in soil culture experiment in our study. Salt stress reduced the growth parameters such as fresh weight, dry weight and branch number of alfalfa seedlings, while ETH treatment alleviated salt stress by improving these growth factors. Salt stress caused leaf yellowing by reducing chlorophyll content, while both ETH and ACC treatments relieved salt stress by increasing chlorophyll contents. It was shown that ethylene receptor was involved in seed germination in *A. thaliana* (Wilson et al., 2014). Knockdown of *MsETR2*, a putative ethylene receptor gene in alfalfa, compromised ethylene mediated tolerance to salt stress in alfalfa plants, demonstrating that the ameliorative effects of ethylene on salt stressed alfalfa plants were dependent on MsETR2. Previously, researchers showed that endogenous hydrogen sulfide (H_2_S) enhanced salt tolerance of alfalfa seedlings (Lai et al., 2014), and exogenous supply of methane (CH_4_) mitigated copper induced inhibition of alfalfa seed germination (Samma et al., 2017). Our results confirmed the importance of another gaseous molecule, ethylene (C_2_H_4_), in improvement of abiotic stress tolerance in alfalfa. ACC, a precursor of ethylene in plants, has the same effects with ethylene but it functions *via* other unknown metabolic pathway, instead of converting to ethylene, as was generally believed before. This indicates that the consensus view on ACC role in other plants is not applicable at least in the plant species like alfalfa. Relief of oxidative stress and decrease in Na^+^ might be the reason for ameliorative effects of ethylene on salt stressed alfalfa plants. However, specific mechanism might exist in a certain variety, while general effects of ethylene were similar in different alfalfa varieties.

## Data Availability Statement

The original contributions presented in the study are included in the article/supplementary material; further inquiries can be directed to the corresponding authors.

## Author Contributions

YW, PD, LK, and RY carried out the experiment and analyzed the data. MZ, TZ and YF performed some of the experiment and contributed to sample preparation. HW and FY designed the study, wrote the manuscript, and supervised the project. YN supervised the experiment of hairy root transformation. All authors contributed to the article and approved the submitted version.

## Funding

This work was supported by the National Natural Science Foundation of China (Grant Nos. 31570142 and 31860055), the innovation guidance project of Inner Mongolia Autonomous Region (Grant No. KCBJ2018001), Science and technology program of Inner Mongolia Autonomous Region (Grant No. 201802061), and Natural Science Foundation of Inner Mongolia Autonomous Region (Grant Nos. 2017MS0330 and 2016bs0319).

## Conflict of Interest

The authors declare that the research was conducted in the absence of any commercial or financial relationships that could be construed as a potential conflict of interest.

## Supplementary Material

Supplementary Figure 1 Effect of different concentrations of NaCl treatments on root length **(A)** and shoot length **(B)** of different alfalfa varieties. ControlK: Water; NaCl: 100, 150, 200, 250 mM. Error bars show the SEM between biological replicates performed (n=3) and Tukey's multiple comparisons test was performed between samples in different groups.

Supplementary Figure 2 Effect of salt stress and mannitol treatment on root length **(A)** and shoot length **(B)** of different alfalfa varieties. ControlK: Water; NaCl: 250 mM. Error bars show the SEM between biological replicates performed (n=3) and Duncan's multiple range test was performed between samples in different groups.

Supplementary Figure 3 Effect of salt stress and mannitol treatment on relative water contents of different alfalfa varieties. ControlCK: Water; NaCl: 250 mM; Mannitol: 100-600 mM. Error bars show the SEM between biological replicates performed (n=3) and Duncan's multiple range test was performed between samples in different groups.

Supplementary Figure 4 Effects of ETH **(A)**, ACC **(B)** and STS **(C)** treatment on germination rates of different alfalfa varieties under salt stress. ControlCK: Water; NaCl: 250 mM. Error bars show the SEM between biological replicates performed (n=3) and Duncan's multiple range test was performed between samples in different groups.

Supplementary Figure 5 Effect of NaCl and combined ethylene treatment on root length **(A)** and shoot length **(B)** of different alfalfa varieties. ControlCK: Water; NaCl: 250 mM; 250 mM NaCl + 0.5, 0.75, 1.0, 1.25, 1.5 mM ETH. Error bars show the SEM between biological replicates performed (n=3) and Duncan's multiple range test was performed between samples in different groups.

Supplementary Figure 6 Effect of ethylene treatment on seedling growths of different alfalfa varieties. ControlCK: Water; ETH: 0.5, 1.0, 1.5 mM. Error bars show the SEM between biological replicates performed (n=3) and Tukey's multiple comparisons test was performed between samples in different groups.

Supplementary Figure 7 Effect of NaCl and combined ethylene treatment on water contents of different alfalfa varieties. ControlCK: Water; NaCl: 250 mM; 250 mM NaCl + 0.5, 0.75, 1.0, 1.25, 1.5 mM ETH. Error bars show the SEM between biological replicates performed (n=3) and Duncan's multiple range test was performed between samples in different groups.

Supplementary Figure 8 Effect of NaCl and combined ACC treatment on seedling growths of different alfalfa varieties. ControlCK: Water; NaCl: 250 mM; 250 mM NaCl + 1, 10, 50, 100 mM ACC. Error bars show the SEM between biological replicates performed (n=3) and Duncan's multiple range test was performed between samples in different groups.

Supplementary Figure 9 Effect of NaCl and combined ACC treatment on water contents of different alfalfa varieties. ControlCK: Water; NaCl: 250 mM; 250 mM NaCl + 1, 10, 50, 100 mM ACC. Error bars show the SEM between biological replicates performed (n=3) and Duncan's multiple range test was performed between samples in different groups.

Supplementary Figure 10 Effect of NaCl and combined STS treatment on seedling growths of different alfalfa varieties. ControlCK: Water; NaCl: 250 mM; 250 mM NaCl + 1, 5, 10, 15 mM STS. Error bars show the SEM between biological replicates performed (n=20) and Duncan's multiple range test was performed between samples in different groups.

Supplementary Figure 11 DAB staining of the seedlings. 1, 8, 15, 22: Water; 2, 9, 16, 23: 250 mM NaCl; 3, 10, 17, 24: 250 mM NaCl+500 μM ETH; 4, 11, 18, 25: 250 mM NaCl+10 μM ACC; 5, 12, 19, 26: 250 mM NaCl+15 μM STS; 6, 13, 20, 27: 250 mM NaCl+500 μM ETH+15 μM STS; 7, 14, 21, 28: 250 mM NaCl+10 μM ACC+15 μM STS. 1~7: Juneng No.2; 8~14: Zhongmu No.3; 9~21: Juneng 418Q; 22~28: Sibeide.

Supplementary Figure 12 Effects of combined treatment on Na^+^ content of different alfalfa varieties under salt stress. ControlCK: Water; NaCl: 250 mM; ETH: 500 μM; ACC: 10 μM; STS: 15 μM. Error bars show the SEM between biological replicates performed (n=10) and Duncan's multiple range test was performed between samples in different groups.

Supplementary Figure 13 Phylogenetic analysis of MsACS (A), MsACO (B) and MsERF (C) homologs from alfalfa.The evolutionary tree was built using the Neighbor-Joining method. All positions with less than 50% site coverage were eliminated. MsACS homolog (LOC25502108), MsACO homolog (LOC11437005), MsERF8 and MsERF11 and used in this study was shown in red diamond.

Supplementary Figure 14 Phylogenetic analysis of MsETR2 with ethylene receptors from different plant species. Construct/Test Neighbor-Joining Tree by MEGA 5.04, Bootstrap method: 1000.

Supplementary Figure 15 The relative expression level of *MsACO* **(A)**, *MsACS* **(B)**, *MsERF8* **(C)** and *MsERF11* **(D)** genes under NaCl and NaCl+ETH treatments after MsETR2 silencing in roots. Roots of control M. sativa and transgenic seedlings were sampled at the seven day post treatment. Relative expression levels of target genes are normalized against steady state levels of alfalfa actin gene.
