# Corrigendum: Ethylene Enhances Seed Germination and Seedling Growth Under Salinity by Reducing Oxidative Stress and Promoting Chlorophyll Content *via* ETR2 Pathway

**DOI:** 10.3389/fpls.2020.639869

**Published:** 2021-01-08

**Authors:** Yue Wang, Pengfei Diao, Lingqi Kong, Ruonan Yu, Man Zhang, Tiantian Zuo, Yanyan Fan, Yiding Niu, Fang Yan, Hada Wuriyanghan

**Affiliations:** ^1^Key Laboratory of Forage and Endemic Crop Biotechnology, Ministry of Education, School of Life Sciences, Inner Mongolia University, Hohhot, China; ^2^State Key Laboratory of Reproductive Regulation & Breeding of Grassland Livestock, School of Life Sciences, Inner Mongolia University, Hohhot, China; ^3^Institute of Grassland Research, Chinese Academy of Agricultural Sciences, Hohhot, China

**Keywords:** alfalfa, salinity, ethylene, *MsETR2*, seed germination, seedling growth

Author contribution for Lingqi Kong was incorrectly written as ^**^Lingqi Kong^3^**. The correct spelling is ^**^Lingqi Kong^3†^**.

The authors apologize for this error and state that this does not change the scientific conclusions of the article in any way. The original article has been updated.

