# Supplementary figures and images for: Ethylene Enhances Seed Germination and Seedling Growth Under Salinity by Reducing Oxidative Stress and Promoting Chlorophyll Content via ETR2 Pathway
Source: Front Plant Sci. 2020 Jul 16;11:1066. doi: 10.3389/fpls.2020.01066 (PMC7378865; doi:10.3389/fpls.2020.01066)

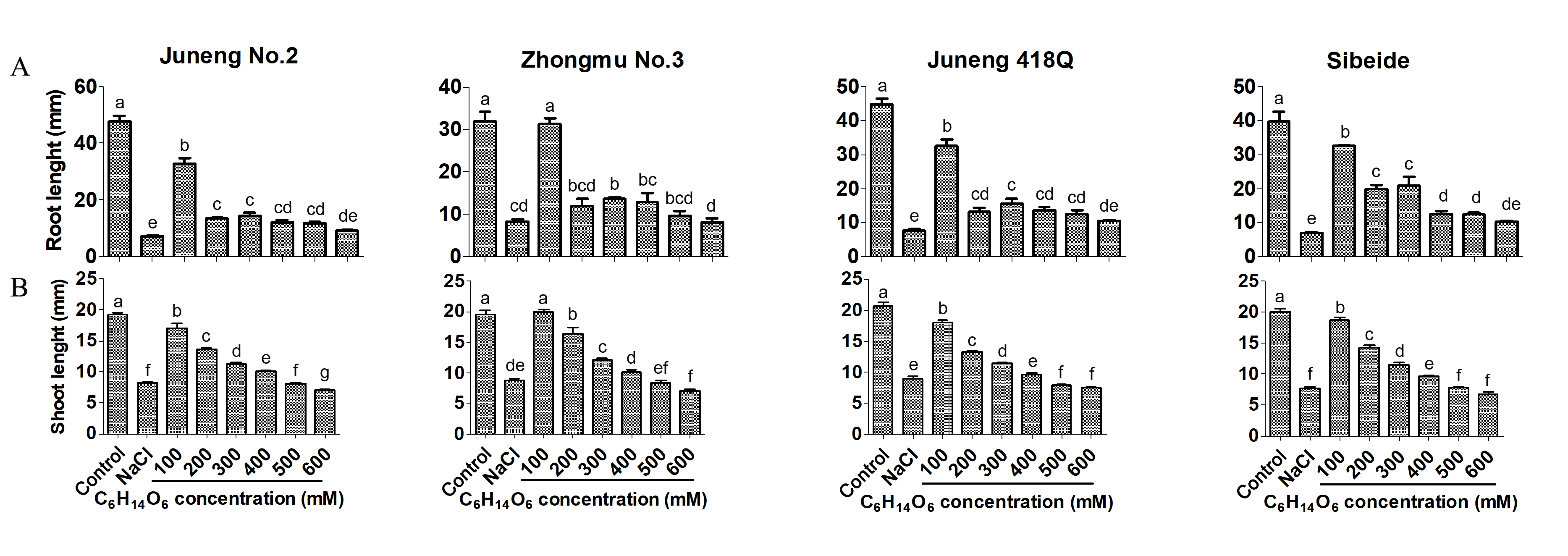

Supplement: Supplementary Figure 2 — Effect of salt stress and mannitol treatment on root length (A) and shoot length (B) of different alfalfa varieties. ControlK: Water; NaCl: 250 mM. Error bars show the SEM between biological replicates performed (n=3) and Duncan's multiple range test was performed between samples in different groups. [file Image_2.tif]

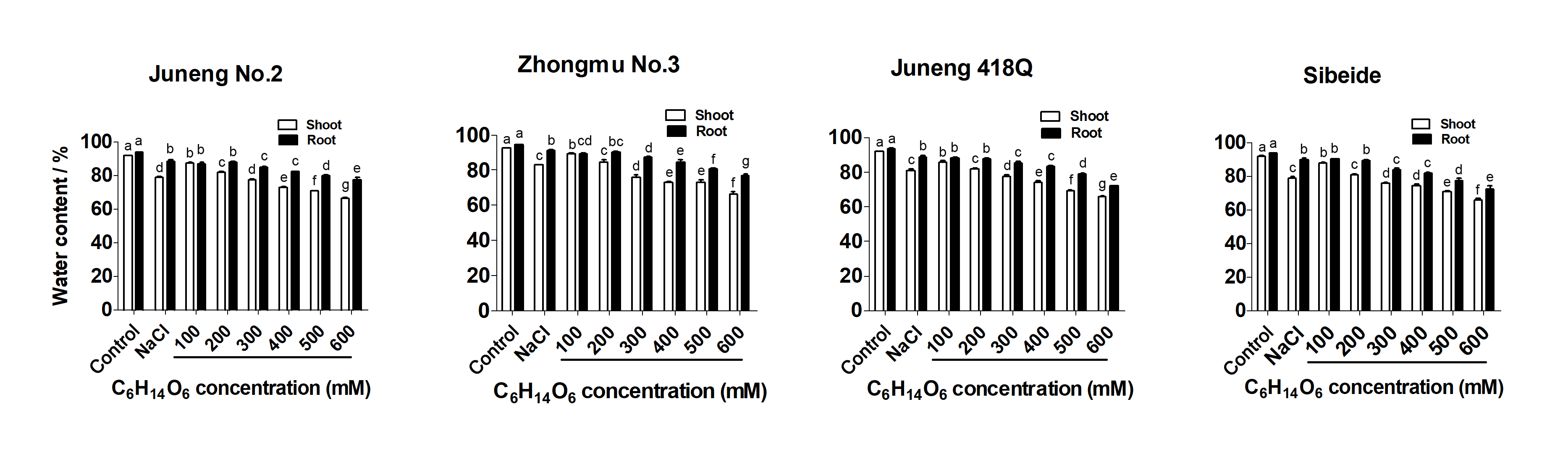

Supplement: Supplementary Figure 3 — Effect of salt stress and mannitol treatment on relative water contents of different alfalfa varieties. ControlCK: Water; NaCl: 250 mM; Mannitol: 100-600 mM. Error bars show the SEM between biological replicates performed (n=3) and Duncan's multiple range test was performed between samples in different groups. [file Image_3.tif]

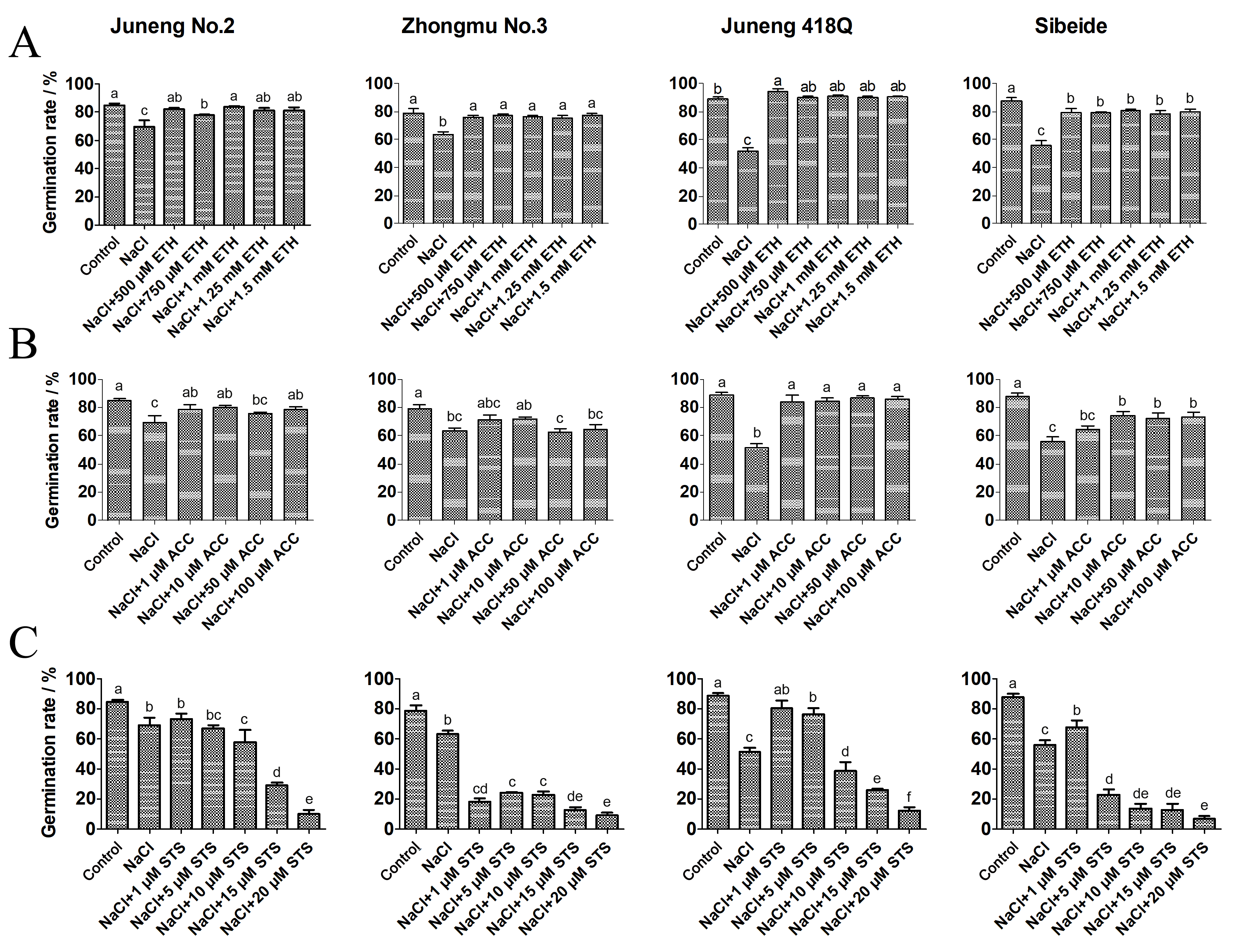

Supplement: Supplementary Figure 4 — Effects of ETH (A), ACC (B) and STS (C) treatment on germination rates of different alfalfa varieties under salt stress. ControlCK: Water; NaCl: 250 mM. Error bars show the SEM between biological replicates performed (n=3) and Duncan's multiple range test was performed between samples in different groups. [file Image_4.tif]

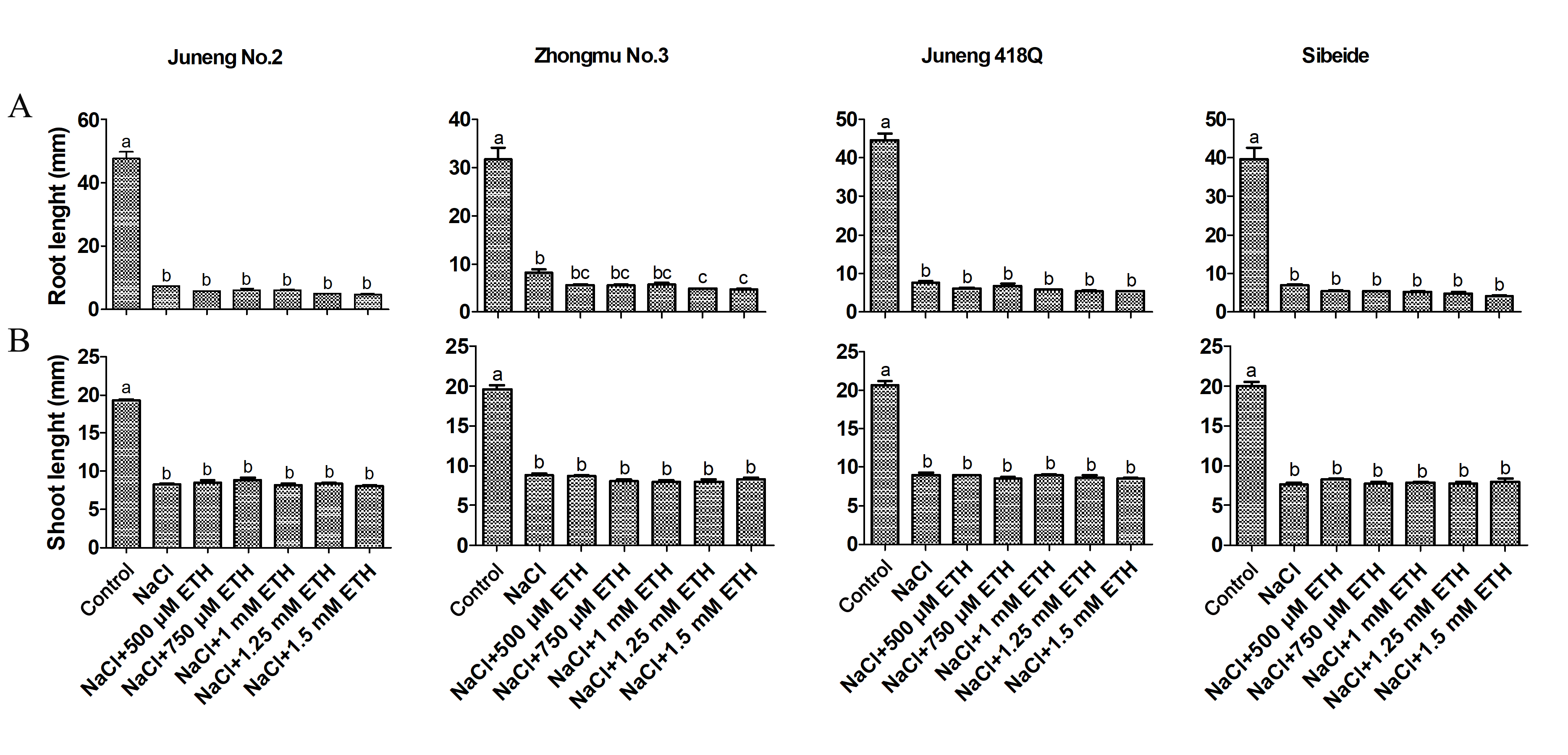

Supplement: Supplementary Figure 5 — Effect of NaCl and combined ethylene treatment on root length (A) and shoot length (B) of different alfalfa varieties. ControlCK: Water; NaCl: 250 mM; 250 mM NaCl + 0.5, 0.75, 1.0, 1.25, 1.5 mM ETH. Error bars show the SEM between biological replicates performed (n=3) and Duncan's multiple range test was performed between samples in different groups. [file Image_5.tif]

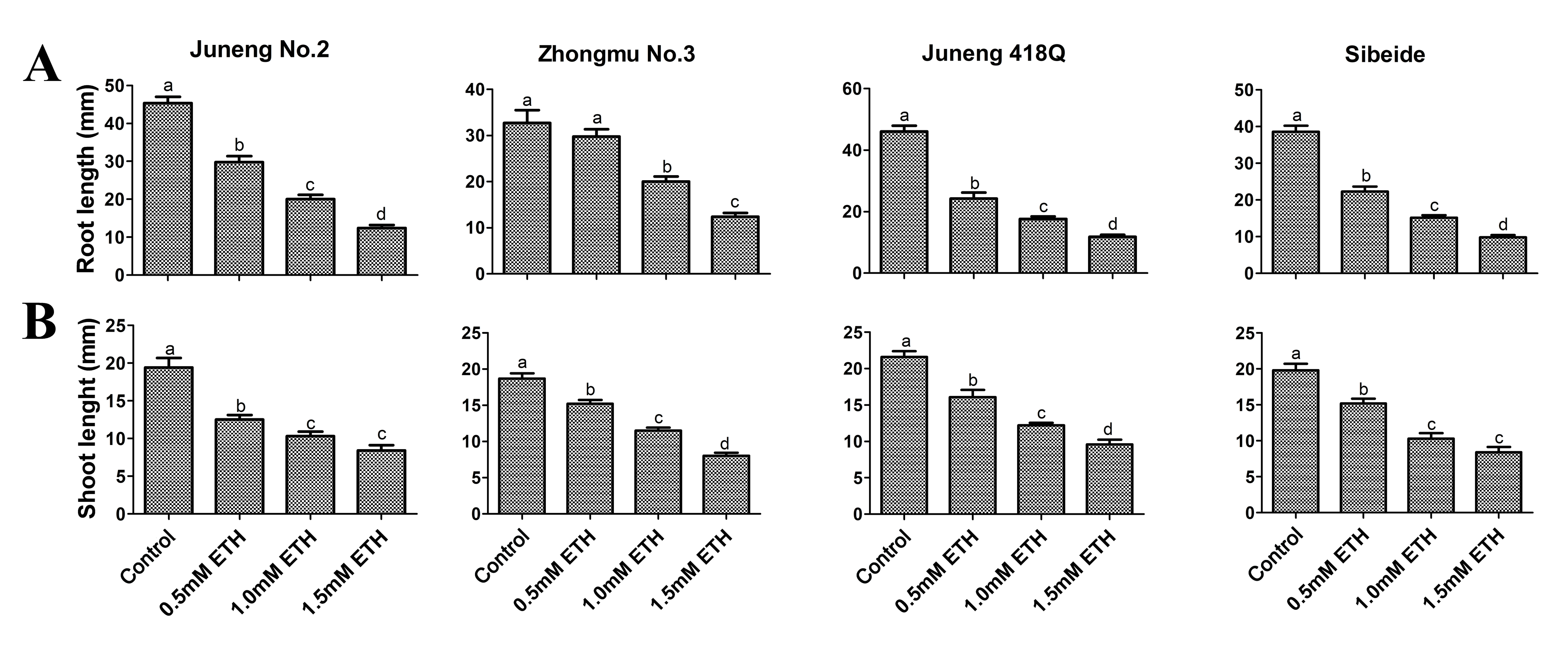

Supplement: Supplementary Figure 6 — Effect of ethylene treatment on seedling growths of different alfalfa varieties. ControlCK: Water; ETH: 0.5, 1.0, 1.5 mM. Error bars show the SEM between biological replicates performed (n=3) and Tukey's multiple comparisons test was performed between samples in different groups. [file Image_6.tif]

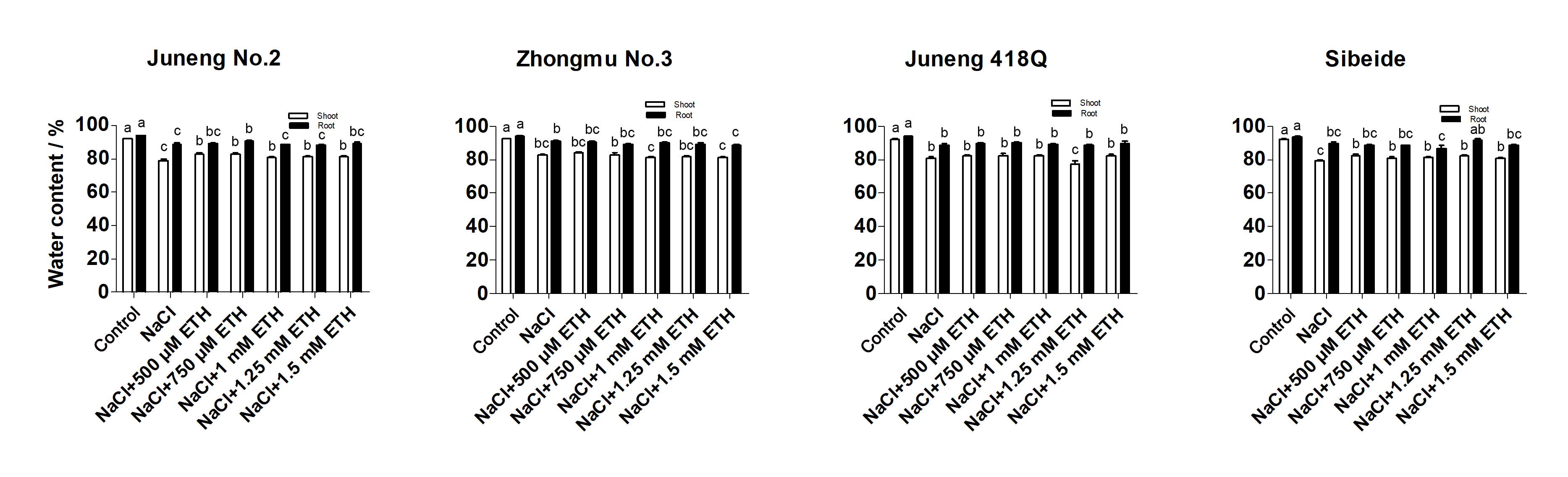

Supplement: Supplementary Figure 7 — Effect of NaCl and combined ethylene treatment on water contents of different alfalfa varieties. ControlCK: Water; NaCl: 250 mM; 250 mM NaCl + 0.5, 0.75, 1.0, 1.25, 1.5 mM ETH. Error bars show the SEM between biological replicates performed (n=3) and Duncan's multiple range test was performed between samples in different groups. [file Image_7.tif]

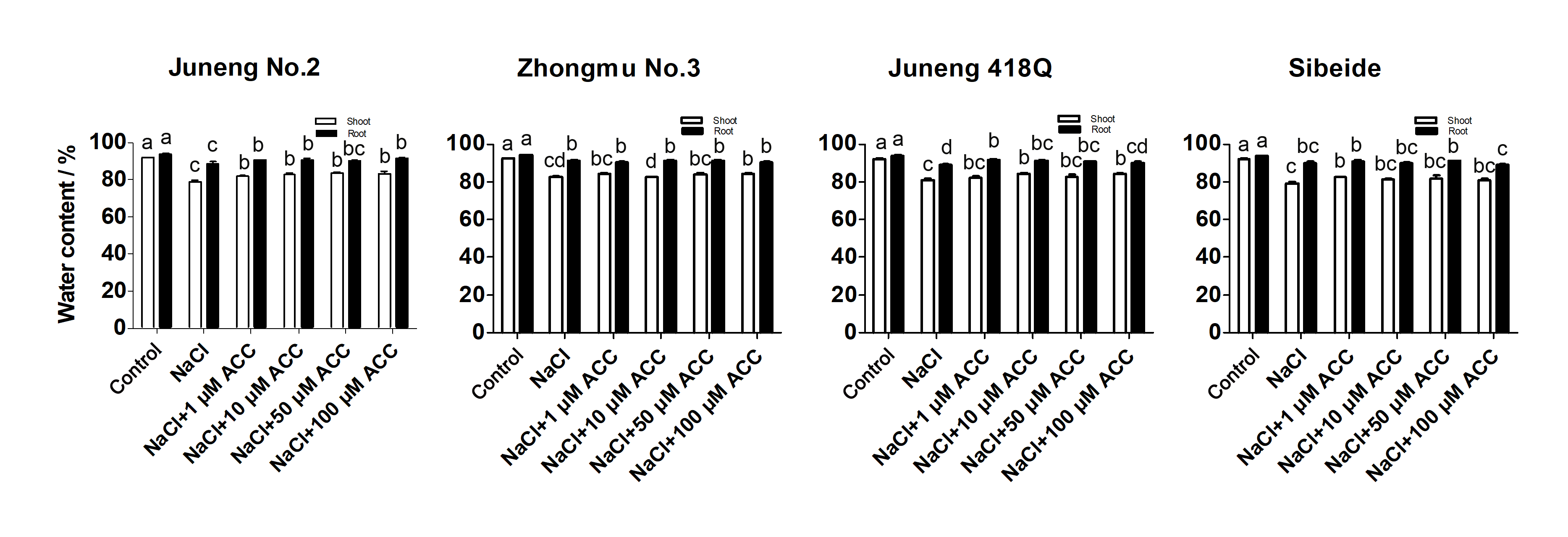

Supplement: Supplementary Figure 8 — Effect of NaCl and combined ACC treatment on seedling growths of different alfalfa varieties. ControlCK: Water; NaCl: 250 mM; 250 mM NaCl + 1, 10, 50, 100 mM ACC. Error bars show the SEM between biological replicates performed (n=3) and Duncan's multiple range test was performed between samples in different groups. [file Image_8.tif]

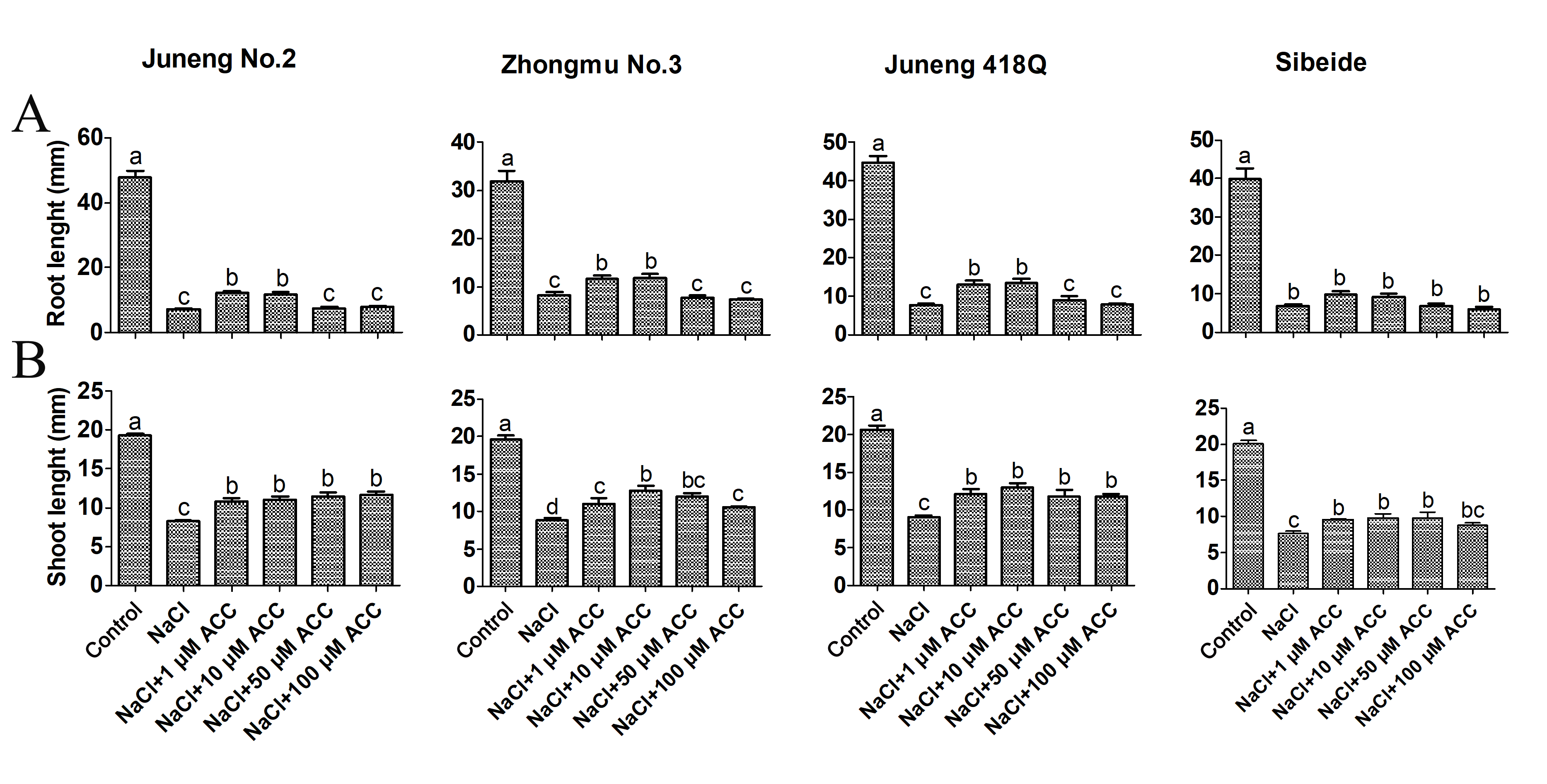

Supplement: Supplementary Figure 9 — Effect of NaCl and combined ACC treatment on water contents of different alfalfa varieties. ControlCK: Water; NaCl: 250 mM; 250 mM NaCl + 1, 10, 50, 100 mM ACC. Error bars show the SEM between biological replicates performed (n=3) and Duncan's multiple range test was performed between samples in different groups. [file Image_9.tif]

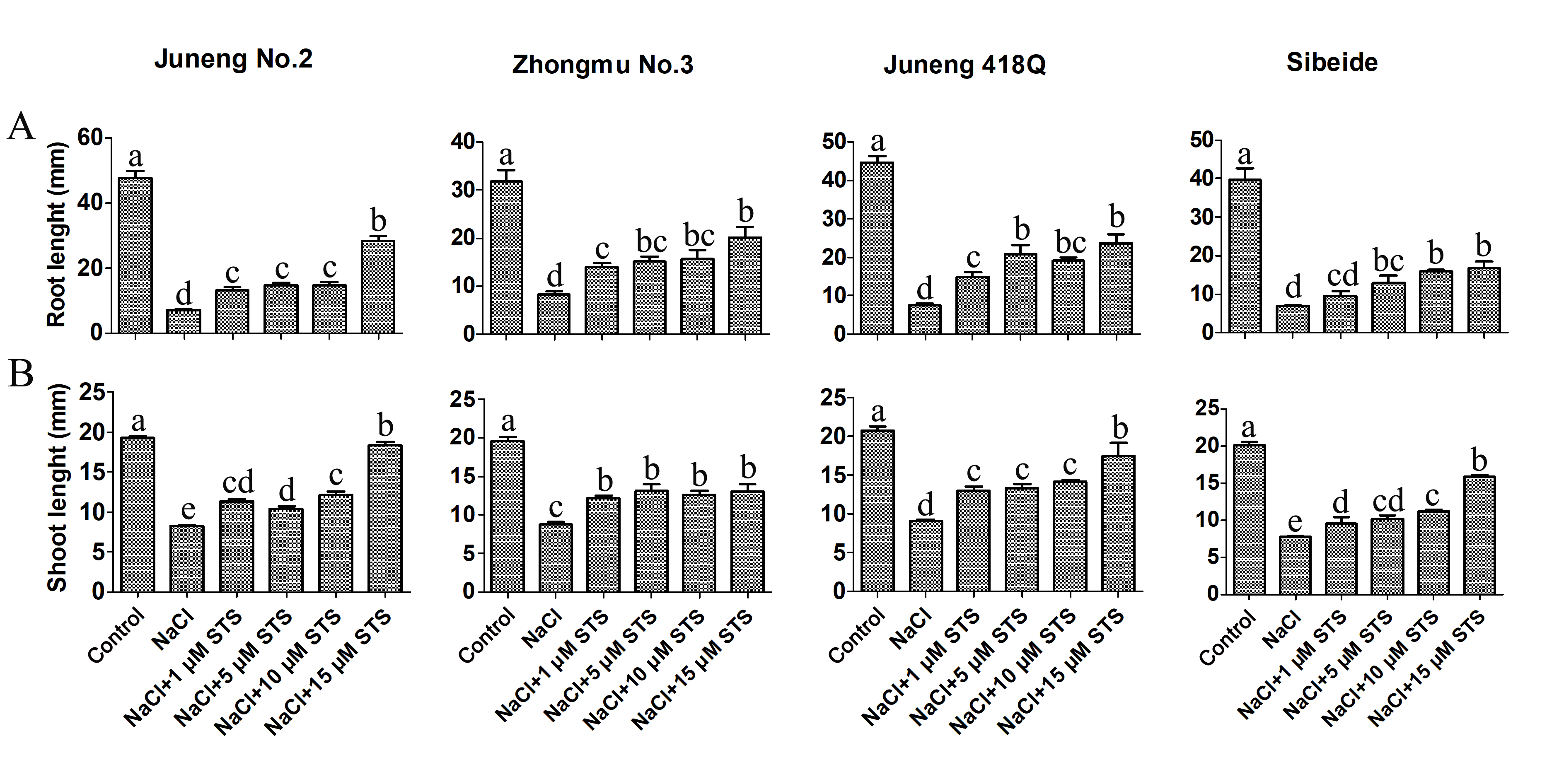

Supplement: Supplementary Figure 10 — Effect of NaCl and combined STS treatment on seedling growths of different alfalfa varieties. ControlCK: Water; NaCl: 250 mM; 250 mM NaCl + 1, 5, 10, 15 mM STS. Error bars show the SEM between biological replicates performed (n=20) and Duncan's multiple range test was performed between samples in different groups. [file Image_10.tif]

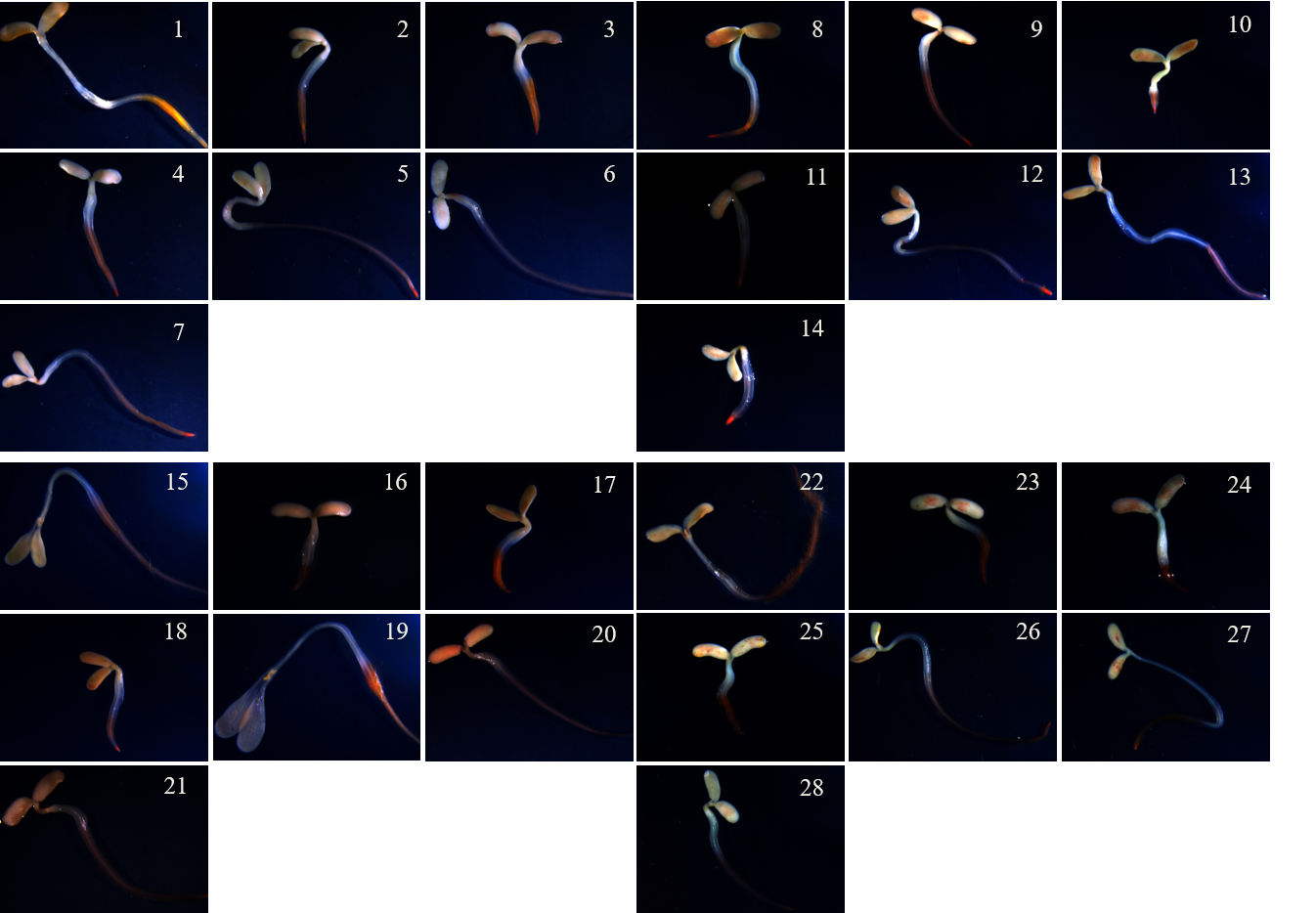

Supplement: Supplementary Figure 11 — DAB staining of the seedlings. 1, 8, 15, 22: Water; 2, 9, 16, 23: 250 mM NaCl; 3, 10, 17, 24: 250 mM NaCl+500 μM ETH; 4, 11, 18, 25: 250 mM NaCl+10 μM ACC; 5, 12, 19, 26: 250 mM NaCl+15 μM STS; 6, 13, 20, 27: 250 mM NaCl+500 μM ETH+15 μM STS; 7, 14, 21, 28: 250 mM NaCl+10 μM ACC+15 μM STS. 1~7: Juneng No.2; 8~14: Zhongmu No.3; 9~21: Juneng 418Q; 22~28: Sibeide. [file Image_11.tif]

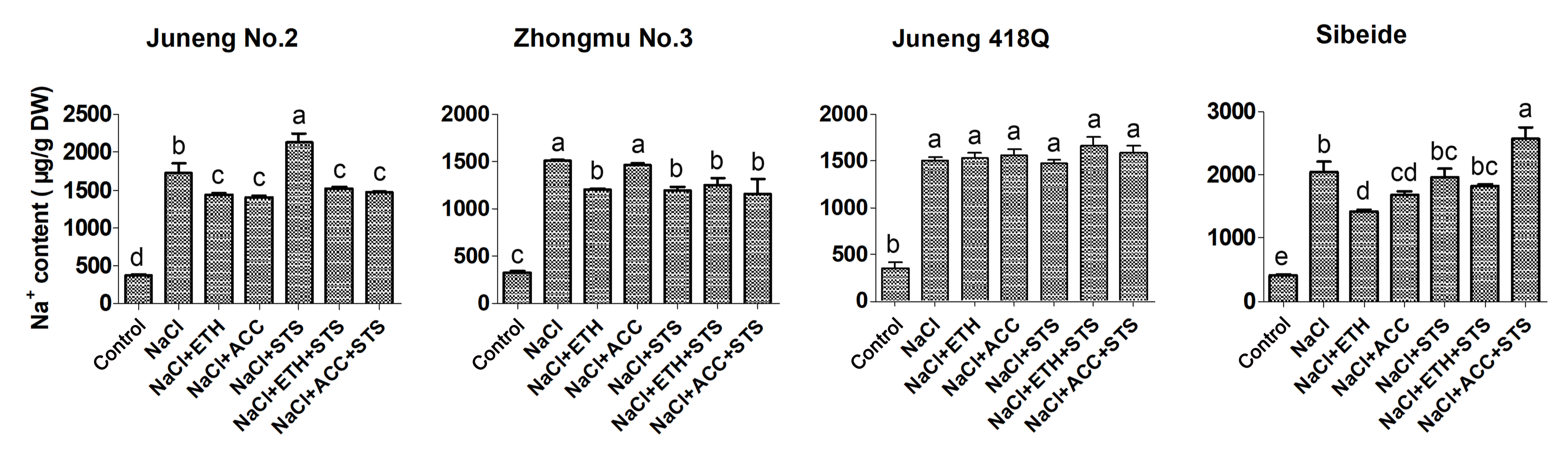

Supplement: Supplementary Figure 12 — Effects of combined treatment on Na+ content of different alfalfa varieties under salt stress. ControlCK: Water; NaCl: 250 mM; ETH: 500 μM; ACC: 10 μM; STS: 15 μM. Error bars show the SEM between biological replicates performed (n=10) and Duncan's multiple range test was performed between samples in different groups. [file Image_12.tif]

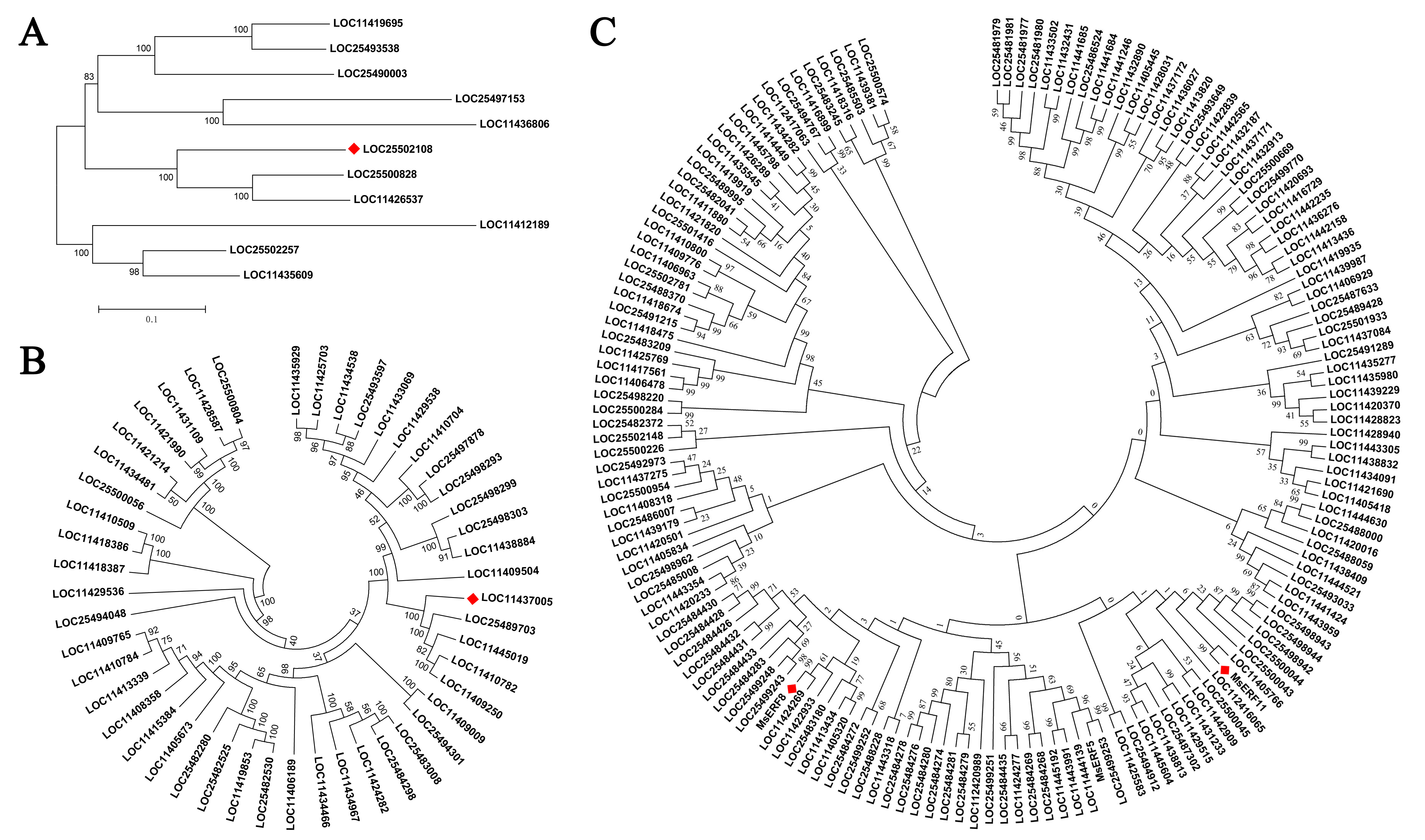

Supplement: Supplementary Figure 13 — Phylogenetic analysis of MsACS (A), MsACO (B) and MsERF (C) homologs from alfalfa.The evolutionary tree was built using the Neighbor-Joining method. All positions with less than 50% site coverage were eliminated. MsACS homolog (LOC25502108), MsACO homolog (LOC11437005), MsERF8 and MsERF11 and used in this study was shown in red diamond. [file Image_13.tif]

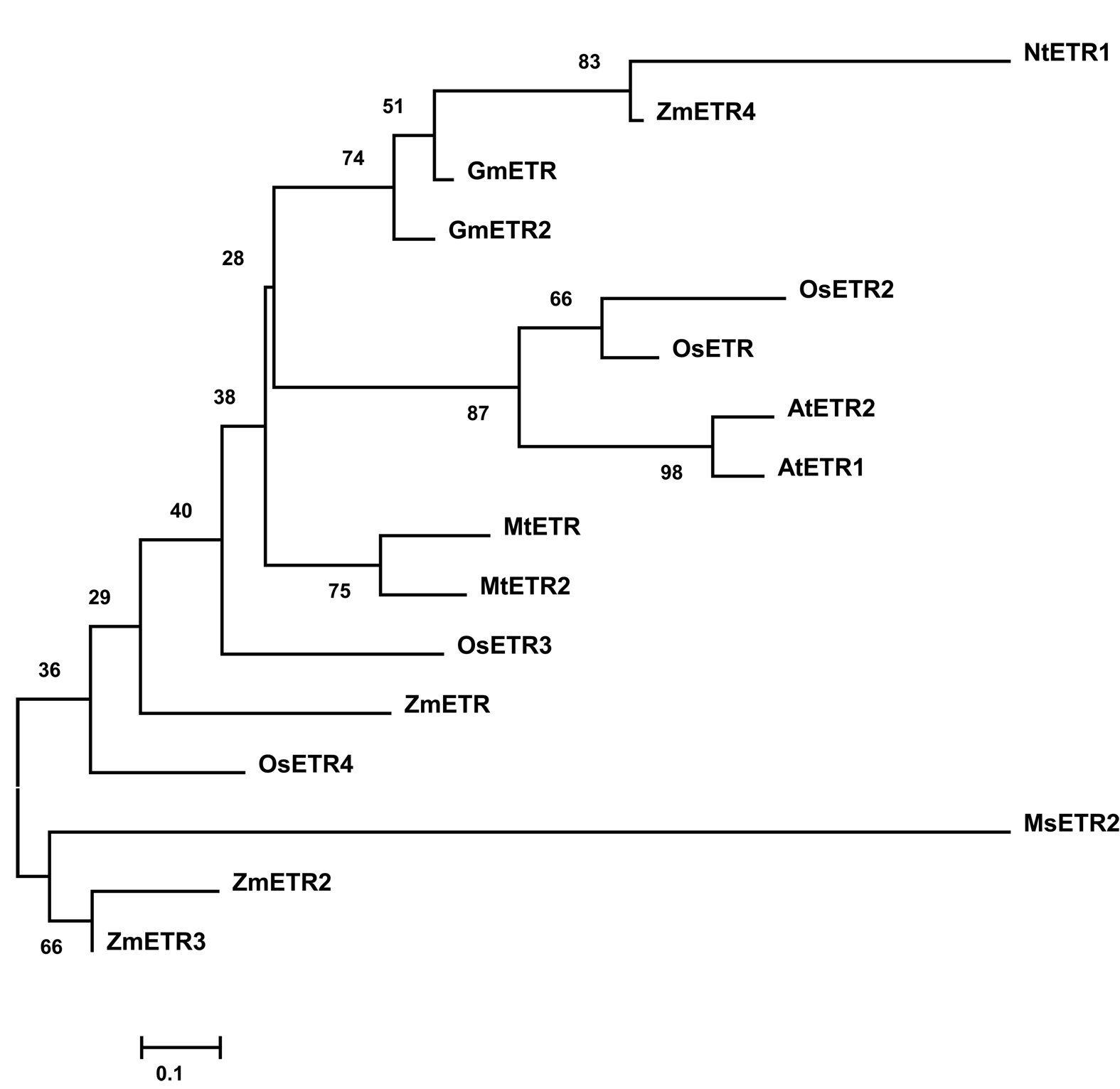

Supplement: Supplementary Figure 14 — Phylogenetic analysis of MsETR2 with ethylene receptors from different plant species. Construct/Test Neighbor-Joining Tree by MEGA 5.04, Bootstrap method: 1000. [file Image_14.tif]

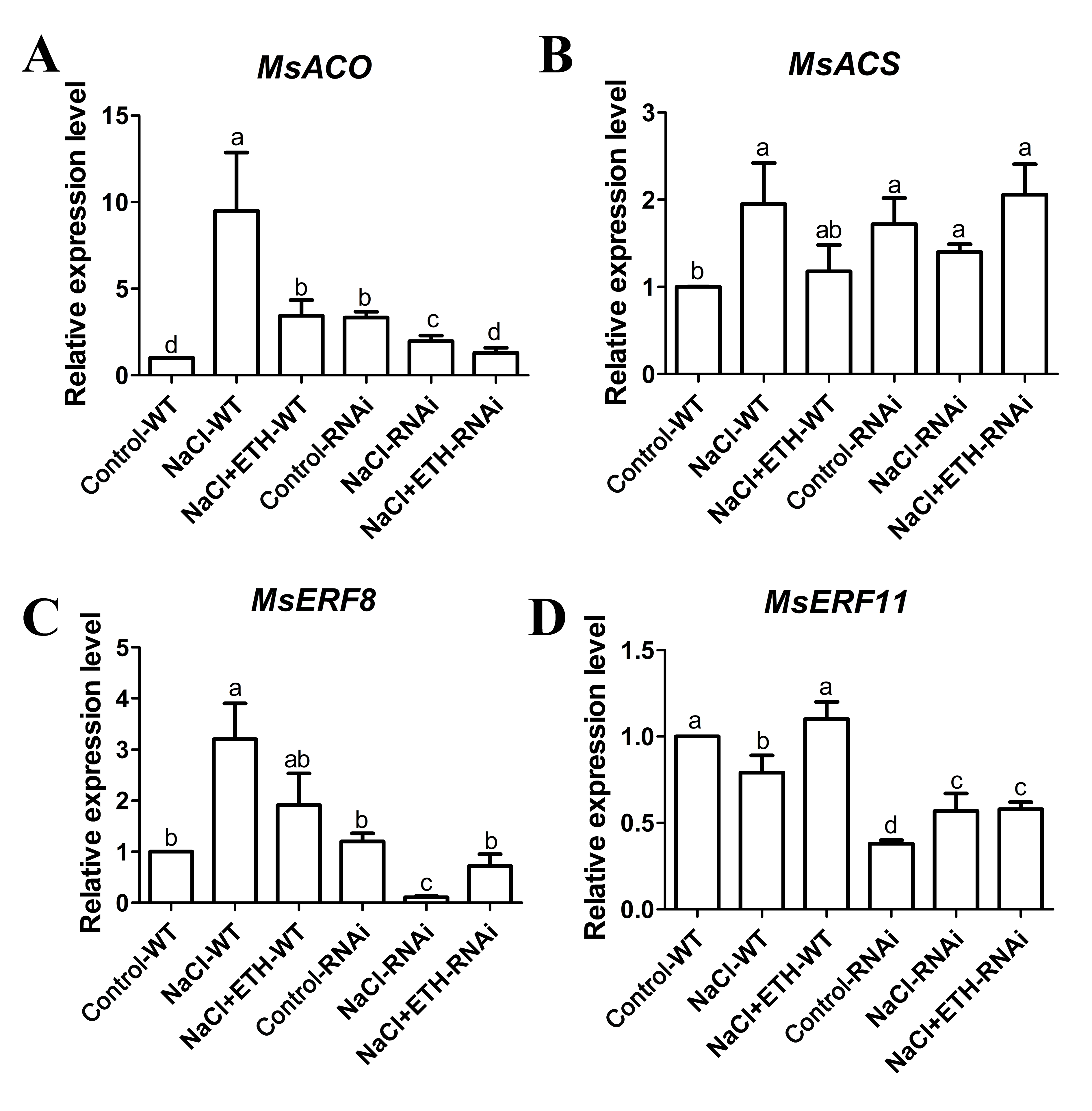

Supplement: Supplementary Figure 15 — The relative expression level of MsACO (A), MsACS (B), MsERF8 (C) and MsERF11 (D) genes under NaCl and NaCl+ETH treatments after MsETR2 silencing in roots. Roots of control M. sativa and transgenic seedlings were sampled at the seven day post treatment. Relative expression levels of target genes are normalized against steady state levels of alfalfa actin gene. [file Image_15.tif]
