# Supplementary material for: Ethylene Enhances Seed Germination and Seedling Growth Under Salinity by Reducing Oxidative Stress and Promoting Chlorophyll Content via ETR2 Pathway
Source: Front Plant Sci. 2020 Jul 16;11:1066. doi: 10.3389/fpls.2020.01066 (PMC7378865; doi:10.3389/fpls.2020.01066)
Supplement: Supplementary file 16 [file Table_1.docx]

**Supplementary Table S1. Primer sequences.**

| Primer | Primer sequence (5’ to 3’) | Purposes |
| --- | --- | --- |
| *MsETR2*-i F | GGAATTCAAGAACTGGAACATTCTGGAAGATCGTCCGTTTATGCCTC | RNAi vector construction |
| *MsETR2*-i R | CGGGATCCTGTGCCTACAGACCCCGTA |  |
| *qMsACO* F | GGGGATTCTTTGAGCTGGTGA | qPCR for *MsACO* |
| *qMsACO* R | ACAGCCTCTAGCCCTTTGGA |  |
| *qMsACS* F | ACGGTGGACTTTTTGTGTGG | qPCR for *MsACS* |
| *qMsACS* R | CCACCCTGGCTCAGAACAAT |  |
| *qMsERF8 F* | TAGAGGTGTAAGGAGAAGGCCA | qPCR for *MsERF8* |
| *qMsERF8* R | CACAAACTCCAGCTTCCAAAGG |  |
| *qMsERF11* F | TCTATTCGCCGCCACTTGTT | qPCR for *MsERF11* |
| *qMsERF11* R | GCGAAGGACGCCGTAAAGTA |  |
| *MsActin* F | CCGACCTCGTCATACTGGTG | qPCR for *MsActin* |
| *MsActin* R | TCTTCAGGAGCAACACGCAA |  |
| *MsETR2*-S F | ctttgtacaaaaaagcaggctcaggggatATGGAAATTCTCCCCTCCGACGAAAC | Subcellular vector construction |
| *MsETR2*-S R | ttgtacaagaaagctgggtgcagggcgatAGTGCCACAAAATGAAATTTTACTGTAC |  |
| *qMsETR2* F | TTTGTACATGTTCGACCGGAA | qPCR for *MsETR2* |
| *qMsETR2* R | TGTGCCTACAGACCCCGTAC |  |
